# Fos ensembles encode and shape stable spatial maps in the hippocampus

**DOI:** 10.1038/s41586-022-05113-1

**Published:** 2022-08-24

**Authors:** Noah L. Pettit, Ee-Lynn Yap, Michael E. Greenberg, Christopher D. Harvey

**Affiliations:** Department of Neurobiology, Harvard Medical School, Boston, MA USA

**Keywords:** Hippocampus, Navigation

## Abstract

In the hippocampus, spatial maps are formed by place cells while contextual memories are thought to be encoded as engrams^1–6^. Engrams are typically identified by expression of the immediate early gene *Fos*, but little is known about the neural activity patterns that drive, and are shaped by, Fos expression in behaving animals^7–10^. Thus, it is unclear whether Fos-expressing hippocampal neurons also encode spatial maps and whether Fos expression correlates with and affects specific features of the place code^11^. Here we measured the activity of CA1 neurons with calcium imaging while monitoring Fos induction in mice performing a hippocampus-dependent spatial learning task in virtual reality. We find that neurons with high Fos induction form ensembles of cells with highly correlated activity, exhibit reliable place fields that evenly tile the environment and have more stable tuning across days than nearby non-Fos-induced cells. Comparing neighbouring cells with and without Fos function using a sparse genetic loss-of-function approach, we find that neurons with disrupted Fos function have less reliable activity, decreased spatial selectivity and lower across-day stability. Our results demonstrate that Fos-induced cells contribute to hippocampal place codes by encoding accurate, stable and spatially uniform maps and that Fos itself has a causal role in shaping these place codes. Fos ensembles may therefore link two key aspects of hippocampal function: engrams for contextual memories and place codes that underlie cognitive maps.

## Abstract (web summary)

Fos-expressing hippocampal neurons form highly correlated ensembles that contribute to spatial coding by forming reliable, long-lasting and spatially unbiased maps of an environment.

## Main

The hippocampus contains engram cells that have a privileged role in the encoding and retrieval of memories^4,12^. Engram cells are typically identified by expression of the activity-inducible transcription factor Fos^13^ during the encoding of a memory^7^, and their synchronous activation can trigger robust memory recall^8,14^. There is growing evidence that neurons defined by Fos expression carry information about context that can be used to form lasting associations, for example, between a specific environment and an aversive outcome. In addition, the hippocampus contains place cells that encode spatial maps of an environment and are thought to support spatial memories and navigation^2,3,15,16^.

Despite roles for engram neurons and place cells in hippocampal memories, remarkably little is known about the relationship between these cells, in part because of the difficulty in relating Fos expression to preceding neural activity patterns during behaviour^5,11,17–21^ and because hippocampal Fos-expressing neurons have not been extensively characterized in typical spatial learning paradigms used to study place cells. We therefore sought to clarify the neural activity patterns that drive Fos expression in the hippocampus of mice performing a spatial memory task.

It is noteworthy that the relationship between neural activity and Fos is bidirectional. Beyond serving as a marker of behaviourally relevant neurons, once induced, Fos acts as a critical regulator of gene expression programmes controlling cellular, synaptic and circuit modifications that in turn fine-tune network activity^11,22–24^. However, while both engram and cognitive map theories indicate that synaptic plasticity occurs to stabilize hippocampal memories^25,26^, few studies have investigated the function of Fos as an inroad to a deeper understanding of the mechanisms of memory formation, consolidation and retrieval.

Towards this goal, we recently demonstrated that Fos orchestrates cell-type-specific inhibitory plasticity in the hippocampal CA1 region and that Fos function is required for hippocampus-dependent spatial learning in the Morris water maze^24^. These results suggest that Fos might modulate place codes, given the known influence of inhibition on place cell firing and the likely role of place cells in spatial learning^16,27,28^. However, it remained to be determined whether Fos in fact has an active role in regulating aspects of place cell function during spatial navigation.

Here we developed a behavioural and imaging approach to study the relationship between Fos expression and place cells. We find that cells with high Fos induction function as ensembles of highly correlated place cells, organized as sequences along a virtual reality track, with reliable activity within their place fields from trial to trial within a session. Moreover, the place fields in these Fos-induced cells uniformly tile the environment, with high across-day stability when compared with non-Fos-induced cells. Using a mouse genetic loss-of-function approach, we further define a causal role for Fos in regulating hippocampal place codes. Cells lacking Fos have place fields with less reliability, spatial selectivity and cross-day stability than their wild-type neighbours. Together, our results indicate that Fos-expressing cells contribute to spatial coding by forming reliable, long-lasting and spatially unbiased maps of an environment and further identify an instructive role for the Fos protein in this process. These findings suggest a link between contextual memories encoded by engrams^4,6^ and cognitive map theories^1^ of hippocampal function.

## Measuring task-related Fos induction

**Fig. 1:**
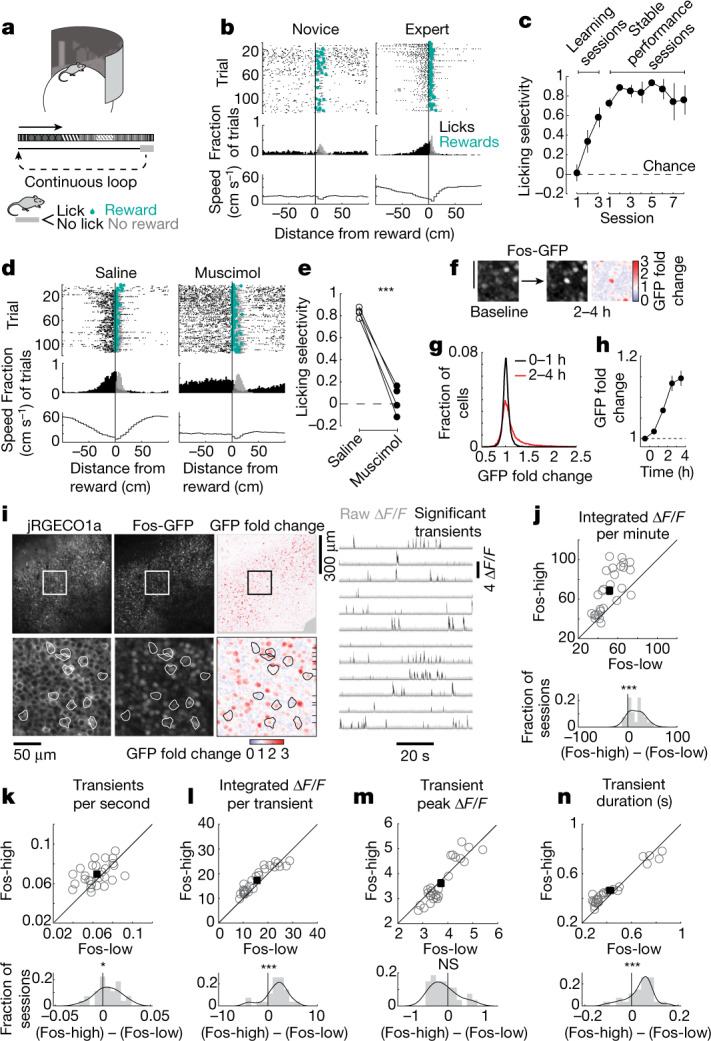
Imaging Fos induction and calcium transients during a learned, hippocampus-dependent spatial navigation task in virtual reality. **a**, Task schematic. **b**, Example behaviour during learning (left) and expert performance (right). Top, raster plot of licks (black), rewards (teal) and reward consumption licks (grey). Middle, fraction of trials with a lick (black) or consumption lick (grey) in each spatial bin. Bottom, average running speed in each spatial bin. **c**, Average learning curve of licking selectivity (Methods). Data (left to right) are shown as the mean ± s.e.m. for *n* = 10, 10, 10, 12, 12, 12, 10, 8, 7, 6 and 5 mice. **d**, Example behaviour following saline (left) or muscimol (right) injection. Similar to **b**. **e**, Licking selectivity for saline and muscimol sessions. Connected dots correspond to individual mice (*n* = 4 mice). *P* < 0.001, two-sided permutation test with 1,000 shuffles. **f**, Measurement of Fos induction, corresponding to the fold change in GFP fluorescence from the start of the session to 2–4 h after the session. Scale bar, 50 µm. **g**, Histogram of fold induction (GFP_after session_/GFP_baseline_) at less than 1 h (black; *n* = 34,359 cells) and 2–4 h (red; *n* = 39,635 cells) from the start of the behaviour session. Of the 73,994 points, 267 beyond the *x*-axis limits are not shown for improved visualization. **h**, Fold induction as a function of time from the start of a behaviour session (1-h time bins). Data are shown as the mean ± s.e.m.; *n* for time points (from left to right) = 37, 35, 2, 19 and 22 sessions. **i**, Representative imaging field of view (left) and calcium traces (right). **j**–**n**, Calcium transient properties of Fos-high versus Fos-low cells. Top, each circle indicates the mean across neurons for one session. Bottom, session-wise difference histogram (grey) with kernel density estimation (black). Two-sided paired-sample *t*  test: *P* = 6 × 10^−6^ (**j**), *P* = 0.047 (**k**), *P* = 0.00066 (**l**), *P* = 0.14 (**m**), *P* = 0.00097 (**n**). *n* = 27 sessions for 6 mice. NS, not significant; *, *P* < 0.05; ***, *P* < 0.001. Source data

Mice running on an air-supported ball traversed a linear 2-m-long track that repeated in a circular topology in virtual reality (Fig. 1a). To receive rewards, mice were required to lick a spout within a 20-cm reward zone (Fig. 1a). We quantified each mouse’s behavioural performance as its licking selectivity, defined by comparing the number of licks near the reward zone with the number of licks in an equally sized portion of the track away from the reward zone. Novice mice licked uniformly across the track and had near-chance licking selectivity (Fig. 1b). Licking selectivity increased over several days as mice learned the location of the reward, after which mice maintained stable, high-selectivity performance (Fig. 1b,c). In all experiments involving the task below, we studied mice that were well trained and thus highly familiar with the task and the environment.

**Extended Data Fig. 1:**
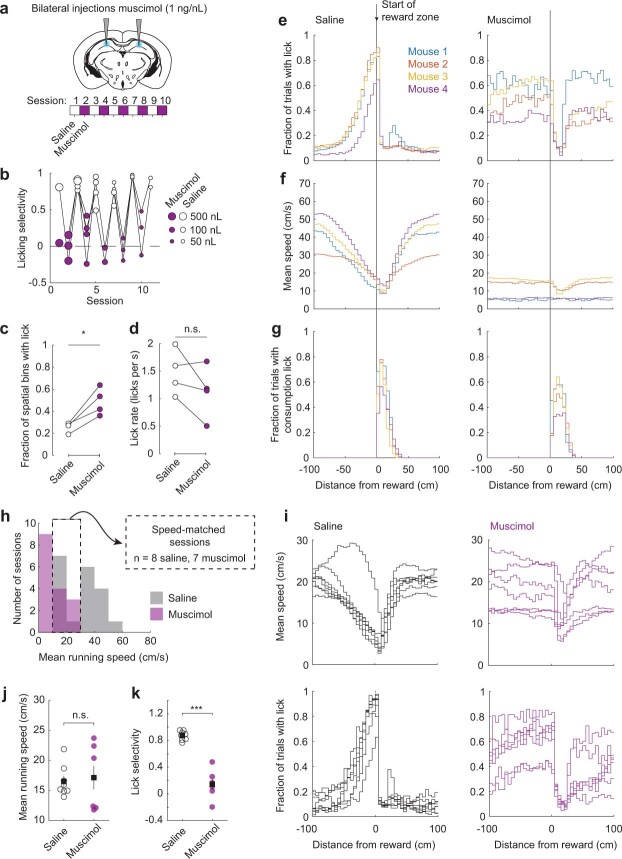
Muscimol-based inactivation of CA1. **a**) Schematic of bilateral injections of muscimol or saline into dorsal CA1. For each mouse, sessions alternated between saline and muscimol across days. Adapted, with permission, from Franklin and Paxinos^79^. **b**) Performance measured as licking selectivity on alternating muscimol and saline sessions. Each set of connected dots is one mouse. n = 4 mice, 8–10 sessions per mouse. **c**) Fraction of spatial bins with a lick for saline and muscimol sessions. Each pair of connected dots is a single mouse. p = 0.019, two-sided permutation test, 1000 shuffles. n = 4 mice, 10 sessions per mouse. **d**) Lick rate for saline and muscimol sessions. Each pair of connected dots is a single mouse. p = 0.17, two-sided permutation test, 1000 shuffles. n = 4 mice, 10 sessions per mouse. **e**–**g**) Fraction of trials with a lick, running speed, and fraction of trials with a consumption lick as a function of maze position for saline and muscimol sessions for individual mice. **h**) Histogram of session average running speed for saline and muscimol sessions. Sessions with mean speeds between 10 and 30 cm/s were chosen for the speed-matched analysis, resulting in 8 saline sessions from 4 mice and 7 muscimol sessions from 2 mice. **i**) Spatially binned running speed and fraction of trials with a lick for speed-matched saline and muscimol sessions. **j**) Mean running speed for speed-matched saline and muscimol sessions. Saline vs. muscimol: p = 0.76; two-sample *t*-test. Error bars, mean ± s.e.m. n = 8 sessions (saline) and 7 sessions (muscimol). **k**) Mean lick selectivity for speed-matched saline and muscimol sessions. Saline vs. muscimol: p = 3.2 x 10^−7^; two-sample *t*-test. Error bars, mean ± s.e.m. n = 8 sessions (saline) and 7 sessions (muscimol). Source data

To test whether hippocampal activity was required for this task, we inactivated CA1 bilaterally by injecting mice with the GABA_A_ (γ-aminobutyric acid type A) receptor agonist muscimol. Muscimol acutely reduced licking selectivity to near-chance levels, whereas injection of saline on neighbouring days did not impair behavioural performance (Fig. 1d,e and Extended Data Fig. 1b,e). Hippocampal inactivation did not appear to affect the engagement or motivation of mice, as mice continued to traverse the track and lick at a similar rate (Extended Data Fig. 1c,d,f,g). Additionally, even though the running speed differed between muscimol and saline sessions, when we subsampled sessions with similar mean running speed, the licking selectivity was still significantly reduced in the muscimol sessions (Extended Data Fig. 1h–k). Therefore, hippocampal activity appears to be necessary for remembering the learned goal location.

**Extended Data Fig. 2:**
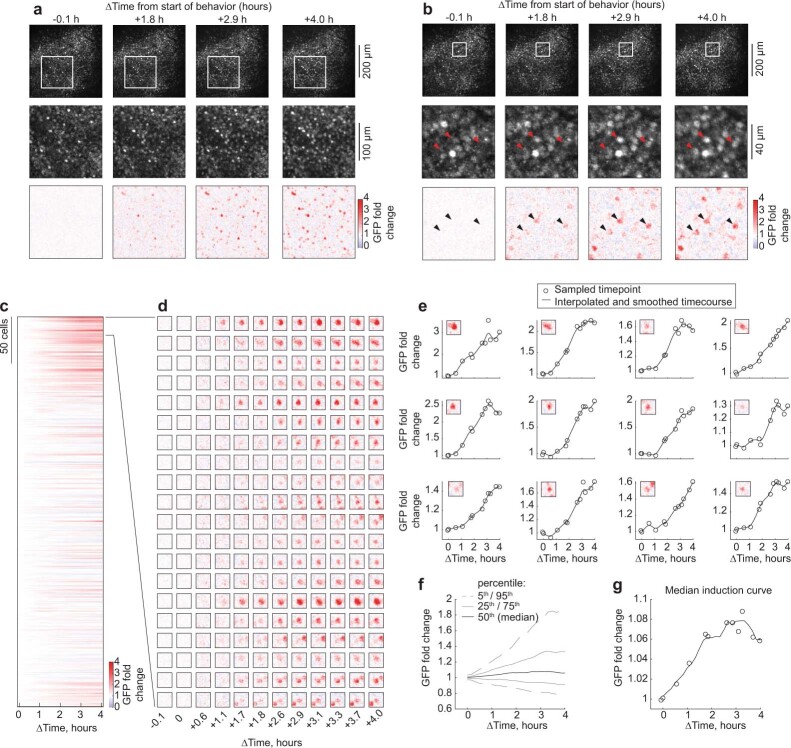
Detailed time course of changes in Fos-GFP expression after behavior. **a**–**b**) GFP fluorescence from the Fos reporter mouse for representative fields-of-view (top) and zoomed in regions (middle) at four timepoints relative to the start of a behavior session. The fold-induction maps (bottom) are shown for the zoomed in areas. Arrows in (**b**) highlight three cells with appreciable increases in GFP fluorescence. **c**) Interpolated and smoothed time courses for the fold-change in GFP fluorescence from the Fos-GFP reporter for 413 cells. **d**) Fold-change images for 20 example cells at various time points relative to the start of the behavior session. **e**) Fold-change values and smoothed time courses for the first 12 cells in (**d**). **f**) Percentiles of the distribution of fold-change values as a function of time from the start of the behavior session. **g**) Median fold-change curve across all cells. n = 413 cells, 1 session, 1 mouse. Source data

**Extended Data Fig. 3:**
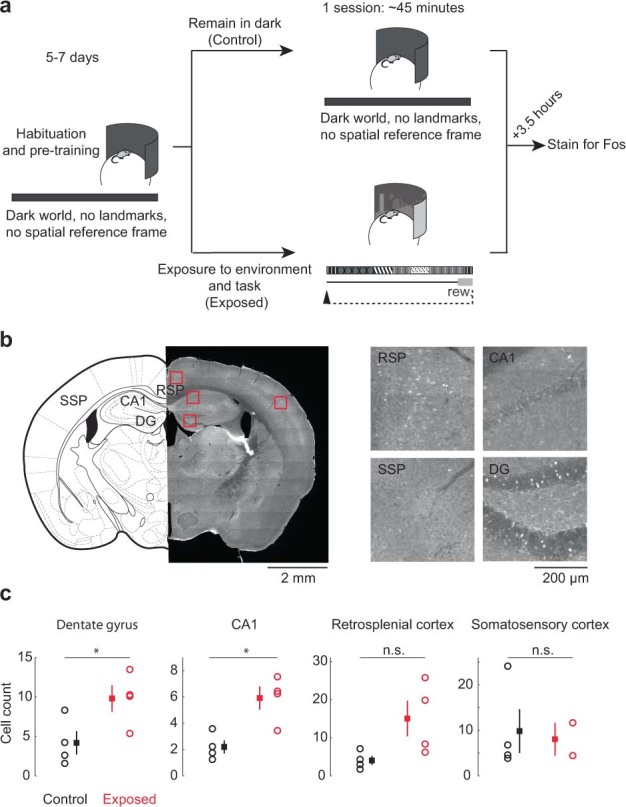
Enrichment of Fos expression in navigation-related areas after virtual navigation. **a**) Schematic of the experiment. Nine mice were habituated to the apparatus and running on the ball for several days. On the day of the experiment, some mice were exposed to a virtual environment and spatial navigation task, while others remained in the dark as on previous days. Approximately 3.5 h after the start of behavior, brains were harvested, followed by staining for Fos expression. **b**) Representative histology images for Fos staining and corresponding atlas plate (reproduced, with permission, from Franklin and Paxinos^79^). Red boxes indicate approximate locations of enlarged image regions on the right. RSP, retrospenial; SSP, somatosensory; DG, dentate gyrus. **c**) Number of Fos-positive cells in 200 x 200 µm regions-of-interest (Methods). Error bars indicate mean ± s.e.m. n = 4 control mice and 4 exposed mice (2 with slices containing SSP). p = 0.046 (DG), p = 0.010 (CA1), p = 0.062 (RSP), p = 0.82 (SSP); two-sided *t*-test. Source data

To monitor changes in Fos expression potentially resulting from the task, we used a *Fos*-transgenic reporter mouse in which enhanced green fluorescent protein (eGFP) with a short half-life is expressed under the control of the *Fos* promoter (hereafter referred to as Fos-GFP)^7^. We quantified Fos induction by comparing the GFP fluorescence of cells before the start of a behavioural session with the fluorescence intensity approximately 3 h later (Fig. 1f–h). We chose this latter time point from a time-course analysis, which showed that fluorescence changes peaked around 3 h after the start of a behaviour session, in line with previous studies using this reporter^29,30^ (Extended Data Fig. 2a–g). In separate experiments using post hoc staining, we observed an increase in Fos expression in the hippocampus of mice exposed to the virtual environment used in our task when compared with expression in mice that ran on a treadmill in darkness (Extended Data Fig. 3a–c). These results indicate that, within CA1, Fos expression may be triggered by navigation through a virtual environment.

## Activity features of Fos-high neurons

**Extended Data Fig. 4:**
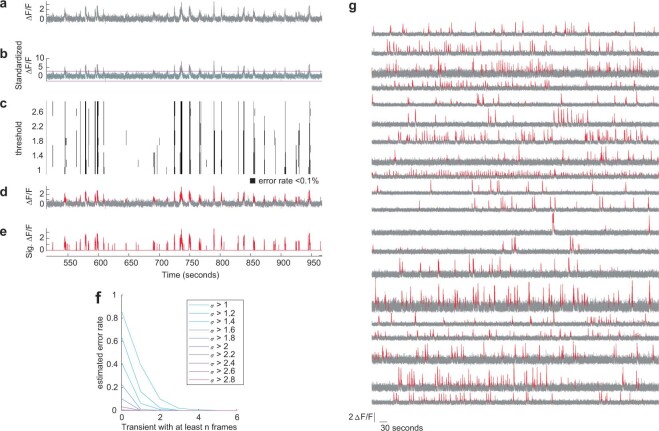
Method for detecting significant calcium transients. **a**) Portion of a representative ∆F/F trace. **b**) The trace from (**a**) normalized and shown with upper (magenta) and lower (blue) σ thresholds for positive-going and negative-going transients. **c**) Binary matrix of significant transient frames for a subset of σ threshold levels. Black indicates significant transients at < 0.1% error rate. Note due to large number of frames in the example, some brief transients are not visible in the rendered image. **d**) Original ∆F/F trace with significant transient timepoints plotted in red. **e**) Significant transient ∆F/F trace with non-significant periods set to zero. **f**) Example error rate as a function of number of frames in the transient, plotted for different threshold levels. **g**) Representative traces for multiple cells with significant transients highlighted in red.

To examine the neural activity patterns that are potentially responsible for induction of Fos, we expressed the red-shifted calcium indicator jRGECO1a in Fos-GFP reporter mice. Using two-photon imaging, we monitored neural activity as calcium transients (Extended Data Fig. 4) in hundreds of CA1 neurons with cellular resolution as mice performed the behavioural task for approximately 30–60 min and quantified GFP expression from the Fos-GFP reporter for each session as described above (Fig. 1f–i). When we refer to neural activity, we are quantifying significant ∆*F*/*F* calcium activity, not spiking or inferred spiking activity (Extended Data Fig. 4 and Methods). Within each session, we compared simultaneously imaged neurons from the same field of view that had differing levels of Fos induction, with a focus on the cells with the 20% highest (Fos-high) and 20% lowest (Fos-low) fold induction. This side-by-side comparison of Fos-high and Fos-low cells from the same session allowed us to eliminate variability in behavioural performance and calcium indicator expression that occurs when comparisons are performed across animals and sessions.

**Extended Data Fig. 5:**
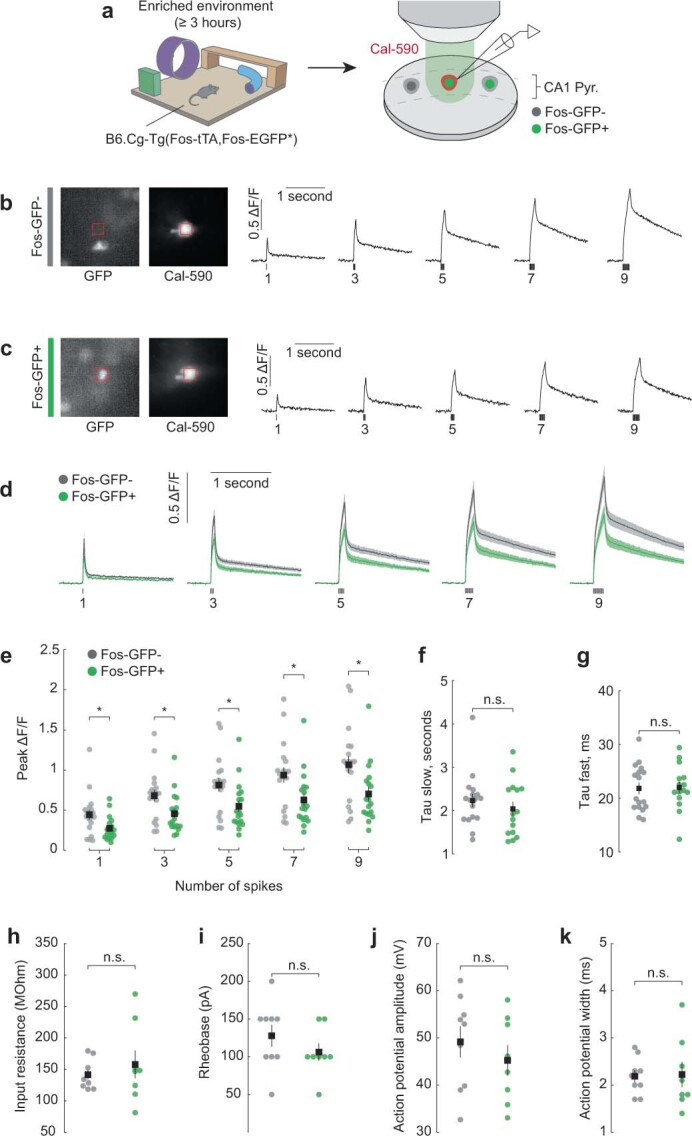
Spike-evoked calcium influx and electrophysiological properties of Fos-expressing neurons. **a**) Schematic of the experiment. Fos reporter mice were exposed to an enriched environment prior to preparation of acute brain slices. **b**–**c**) Left, example images and ∆F/F fluorescence traces for Fos-GFP- and Fos-GFP+ cells. Red boxes are somatic ROIs used to extract fluorescence traces. Right, example calcium imaging traces. Black ticks mark evoked spikes, numbers indicate number of spikes in a 50 Hz train. **d**) Average spike-evoked ∆F/F fluorescence for 1, 3, 5, 7, and 9 action potentials. Fos-GFP-: n = 19 cells, 6 mice. Fos-GFP+: n = 18 cells, 6 mice. Shading, mean ± s.e.m. **e**) Quantification of peak calcium influx. Fos-GFP- vs. Fos-GFP+: 1 AP, p = 0.021; 3 APs, p = 0.020; 5 APs, p = 0.020; 7 APs, p = 0.017; 9 APs p = 0.013; two-sample *t*-test. Fos-GFP-: n = 19 cells, 6 mice. Fos-GFP+: n = 18 cells, 6 mice. Black squares and error bars denote mean ± s.e.m. **f**–**g**) Decay kinetics quantified with double-exponential fit. Fos-GFP- vs. Fos-GFP+: tau slow, p = 0.40; tau fast, p = 0.91; two-sample *t*-test. Fos-GFP-: n = 17 cells, 5 mice. Fos-GFP+: n = 15 cells, 5 mice. 5 cells were excluded due to poor double-exponential fit. Black squares and error bars denote mean ± s.e.m. **h**–**k)** Quantification of electrophysiological properties. Fos-GFP- and Fos-GFP+: input resistance, p = 0.48; rheobase, p = 0.27; AP amplitude, p = 0.41; AP width, p = 0.90; two-sample *t*-test: Fos-GFP-: n = 9 cells, 1 mouse; Fos-GFP+: n = 8 cells, 1 mouse. Black squares and error bars denote mean ± s.e.m. Source data

Fos-high cells had larger integrated ∆*F*/*F* values, and thus larger total calcium influx, relative to Fos-low cells (Fig. 1j), in line with the known role of increased calcium influx in driving gene transcription through voltage-sensitive calcium channels^31–33^. The larger calcium influx in Fos-high cells did not arise from a substantially greater number of calcium transients per second but rather resulted mainly from higher integrated calcium levels per transient (Fig. 1k,l). The higher calcium influx per transient was due to longer transients rather than a change in the amplitude of transients (Fig. 1m,n). Because previous work has shown that calcium transients in pyramidal neuron cell bodies are related to spiking^34–36^, these findings may reflect different spiking patterns in Fos-high and Fos-low cells^10^. However, the relationship between spiking and calcium transients may also be different for these groups of cells. To test the latter possibility, we compared the spike-evoked calcium influx in cells with and without Fos induction using current-clamp recordings and calcium imaging in brain slices (Extended Data Fig. 5a–c). Cells with Fos induction had smaller calcium transients in response to triggered action potentials, a finding that warrants future investigation. The kinetics of calcium transients and intrinsic electrophysiological properties were similar to those in cells without Fos induction (Extended Data Fig. 5d–k). Therefore, Fos-high cells have higher total calcium influx during the navigation task and Fos-expressing cells have lower calcium influx per spike ex vivo, suggesting that Fos-induced cells may have more spiking or a different pattern of spiking than non-Fos-induced cells. Although we could not resolve the precise underlying in vivo spiking patterns associated with Fos induction, our results indicate that prolonged elevated calcium may have an important role in inducing the expression of Fos during a navigation task.

## Robust place coding in Fos-high neurons

**Fig. 2:**
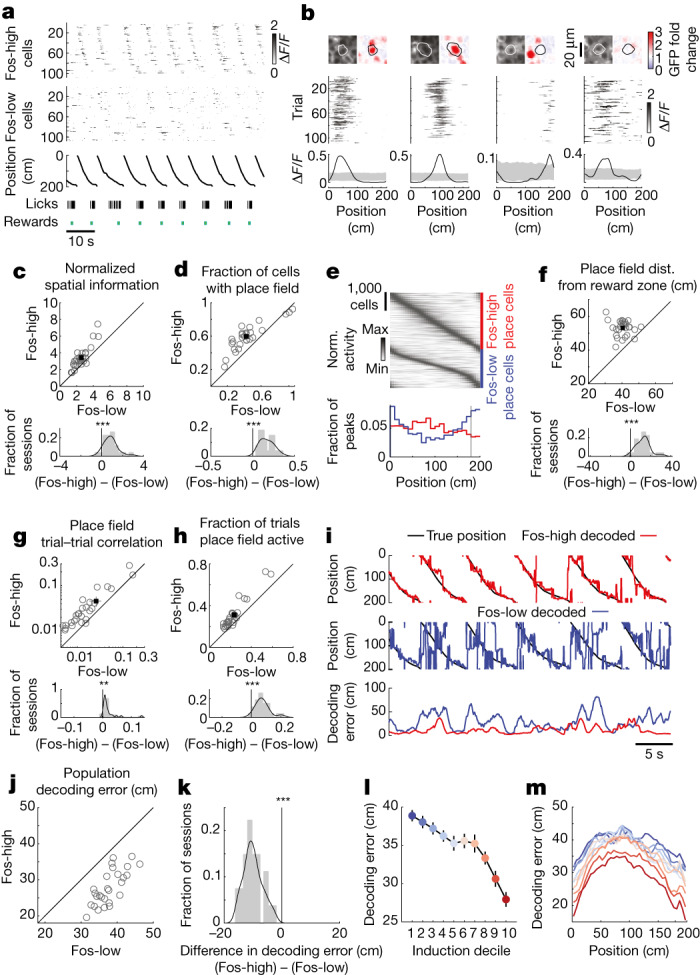
Relationship between Fos induction and the place coding properties of CA1 neurons. **a**, Example population activity of simultaneously recorded Fos-high (top) and Fos-low (middle) cells. Bottom, position along the track, licks (black) and rewards (teal). **b**, Representative Fos-high (left) and Fos-low (right) cells with spatially modulated activity. Top, jRGECO1a and fold-induction map images with a smoothed cell contour drawn. Middle, trial-wise spatially binned activity. Bottom, mean spatially binned activity, smoothed with a Gaussian kernel (s.d., 10 cm). Grey shading indicates the 1st and 99th percentiles from permutations of calcium time series relative to behaviour time series. **c**,**d**, Properties of simultaneously imaged Fos-high (*y* axis) and Fos-low (*x* axis) populations. Top, each circle indicates the mean across neurons for one session. Bottom, difference histogram (grey) with kernel density estimation (black). Two-sided paired-sample *t*  test: *P* = 6.7 × 10^−7^ (**c**), *P* = 2.9 × 10^−11^ (**d**). *n* = 27 sessions for 6 mice. **e**, Normalized activity of Fos-high (top) and Fos-low (middle) place cells as a function of track position. Bottom, histogram of peak location for Fos-high (red) and Fos-low (blue) cells. The vertical grey line indicates the reward site. **f**–**h**, Similar to **c**,**d**. Only cells with significant place fields are included. Two-sided paired-sample *t*  test: *P* = 9.6 × 10^−10^ (**f**), *P* = 0.001 (**g**), *P* = 2.4 × 10^−8^ (**h**). **i**, Example naive Bayes’ decoding of position from activity in Fos-high versus Fos-low populations. **j**,**k**, Scatterplot and difference histogram as in **c** showing the decoder error for Fos-high versus Fos-low populations. Two-sided paired-sample *t*  test: *P* = 5.0 × 10^−15^. *n* = 27 sessions for 6 mice. **l**, Average decoder error as a function of induction decile. Data are shown as the mean ± s.e.m.; *n* = 27 sessions for 6 mice. **m**, Mean error as a function of position on the track, plotted for each induction decile. **, *P* < 0.05; ***, *P* < 0.001. Source data

**Extended Data Fig. 6:**
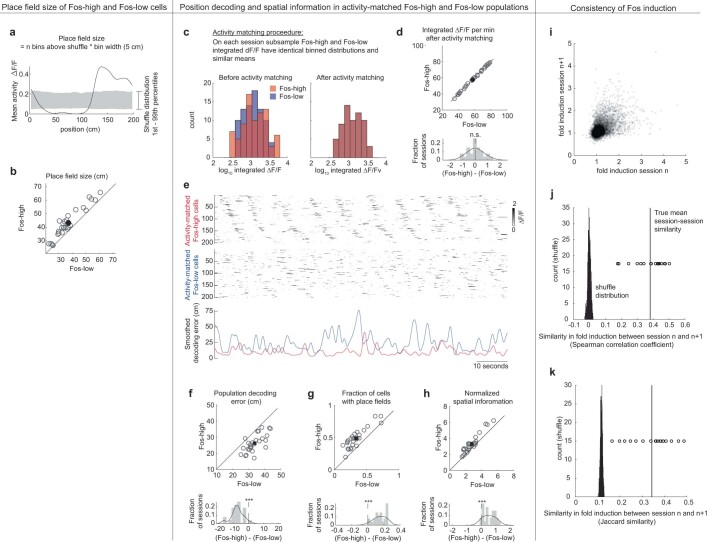
Place field size of Fos-high and Fos-low cells, position decoding and spatial information in activity-matched Fos-high and Fos-low populations, and consistency of Fos induction across stable performance sessions. **a**) Example place field and description of size measurement. Place field size is quantified as the number of bins above the 99^th^ percentile of the shuffle distribution, multiplied by the width of each bin. **b**) Scatter of each session’s average place field size for simultaneously recorded Fos-high and Fos-low cells. Fos-high vs. Fos-low: p = 1.1 x 10^−9^, two-sided paired-sample *t*-test; n = 27 sessions, 6 mice. **c**) Activity matching procedure and example session. **d**) Session-wise scatter and difference histogram of mean integrated ∆F/F per minute in Fos-high and Fos-low populations. After activity-matching, Fos-high vs. Fos Low: p = 0.85, two-sided paired-sample *t*-test. n = 27 sessions, 6 mice. **e**) Example activity and decoding performance on held-out test data from activity-matched Fos-high and Fos-low populations. Not all timepoints are necessarily contiguous as train-test split used interleaved trials (Methods). Top and middle: activity of Fos-high and Fos-low cells. Each row is a different cell, and cells are sorted by their preferred position on the track. Bottom: smoothed (gaussian kernel, 0.5 s standard deviation) absolute frame-wise decoding error for Fos-high (red) and Fos-low (blue) decoders. **f**) Session-wise scatter and difference histogram comparing decoding error. Fos-high vs. Fos-low: p = 5.2 x 10^−10^, two-sided paired-sample *t*-test. n = 27 sessions, 6 mice. **g**) Session-wise scatter and difference histogram comparing fraction of cells with place fields. Fos-high vs. Fos-low: p = 4.9 x 10^−11^, two-sided paired-sample *t*-test. n = 27 sessions, 6 mice. **h**) Session-wise scatter and difference histogram comparing decoding error. Fos-high vs. Fos-low: p = 1.4 x 10^−7^, two-sided paired-sample *t*-test. n = 27 sessions, 6 mice. **i**) Scatter of fold induction values in cells recorded on consecutive sessions. n = 12,645 cells, 18 sessions, 4 mice. **j**) Similarity in Fos fold induction between session n and session n+1, measured as the Spearman correlation coefficient. Circles indicate the mean for individual sessions, and the vertical line indicates the mean across sessions. Histogram indicates the mean similarity in 1000 permutations of cell identities. p < 0.001. n = 18 sessions from 4 mice. **k**) Similar to (**j**), except with similarity in Fos induction measured as Jaccard similarity. p < 0.001. Source data

Next, we asked whether there are differences in the place coding properties of Fos-high and Fos-low neurons. As expected, during the task, many of the cells in CA1 had activity that was significantly modulated across the track and formed place fields (Fig. 2a,b). Although place cells could be found in both the Fos-high and Fos-low populations, Fos-high cells were more likely to be place cells than Fos-low cells (Fig. 2d). Fos-high cells also had higher spatial information than Fos-low cells, measured as the normalized mutual information between a cell’s activity and the mouse’s location, which is independent of our place field definition (Fig. 2c,d). Fos-high place fields tended to be wider than Fos-low place fields (Extended Data Fig. 6a,b). Notably, Fos-high and Fos-low place cells exhibited distinct distributions of their place fields across the track, with Fos-low place fields enriched near the reward zone and Fos-high place fields mostly uniformly distributed across the track and even showing a slight bias away from the reward zone (Fig. 2e,f). Fos-high cells also had more reliable calcium transients from trial to trial, measured as the correlation in activity for pairs of trials during a session (Fig. 2g). Moreover, Fos-high cells had activity within their place field on a larger fraction of trials than Fos-low cells (Fig. 2h).

Given the higher fraction of cells with place fields and higher reliability in activity for Fos-high cells, we reasoned that these cells may constitute a subpopulation well suited for representing the mouse’s current location. We trained a naive Bayes’ decoder to predict the mouse’s location on the basis of the activity in a population of Fos-high or Fos-low neurons (Fig. 2i). In all sessions, the accuracy of decoding the mouse’s position using a population of Fos-high cells was greater than with an equally sized population of Fos-low cells, observed as lower decoding error in the Fos-high population (Fig. 2j,k). This improvement in decoding with higher Fos induction was present across all spatial positions of the track and increased with the level of Fos induction (Fig. 2l,m). To test whether the increased decoding accuracy could be explained by higher calcium activity in the Fos-high population, we subsampled Fos-high and Fos-low cells in each session to create populations with similar integrated ∆*F*/*F* (Extended Data Fig. 6c,d). Even after matching activity levels, Fos-high cells exhibited significantly lower decoding error, higher incidence of place fields and greater spatial information than their Fos-low counterparts (Extended Data Fig. 6e–h). Thus, Fos-high cells have a link to place coding that goes beyond their higher calcium activity.

**Extended Data Fig. 7:**
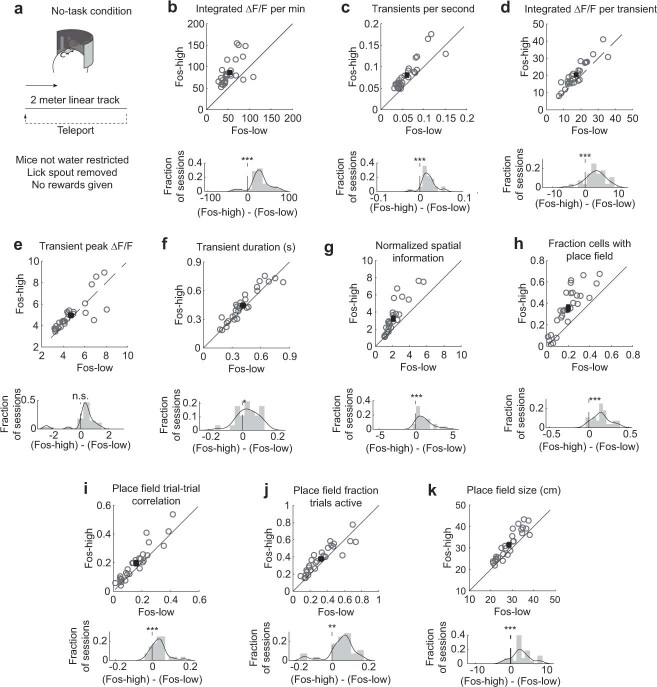
Calcium activity and place coding properties of Fos-high and Fos-low cells during virtual navigation in the absence of a task. **a**) No-task condition schematic and description. **b**–**f**) Properties of calcium transients for simultaneously recorded Fos-high and Fos-low cells. Top: Each circle indicates the mean across neurons for one session. Bottom: Histogram of the difference between Fos-high and Fos-low cells across sessions (gray) with the kernel density estimation (black). For panels (**c**–**d**), only Fos-high and Fos-low cells with significant place fields are included. **g**–**k**) Spatial coding properties of simultaneously recorded Fos-high and Fos-low cells. Top: Each circle indicates the mean across neurons for one session. Bottom: Histogram of the difference between Fos-high and Fos-low cells across sessions (gray) with the kernel density estimation (black). Fos-high vs. Fos-low cells: p = 1.2 x 10^−7^ (**b**), p = 3.8 x 10^−7^ (**c**), p = 8.6 x 10^−6^ (**d**), p = 0.16 (**e**), p = 0.013 (**f**), p = 5.1 x 10^−6^ (**g**), p = 6.5 x 10^−8^ (**h**), p = 0.00015 (**i**), p = 0.0019 (**j**), p = 4.2 x 10^−6^ (**k**); two-sided paired-sample *t*-test. n = 28 sessions, 5 mice. Source data

These results indicate that Fos-high cells collectively form a spatial map of the environment. We performed two additional experiments to test this idea. First, we asked whether Fos-high cells are still related to a spatial map in the absence of a behavioural task. A separate cohort of mice ran through virtual environments but without performing a spatial learning task or receiving rewards. In these experiments, a place code is still present but other task-related factors that could drive Fos expression are absent, such as reward. The differences between Fos-high and Fos-low cells were qualitatively identical to those in mice trained on the task: Fos-high cells had significantly higher spatial information, a greater prevalence of place fields, more reliable place field activity and higher integrated calcium levels (Extended Data Fig. 7).

**Extended Data Fig. 8:**
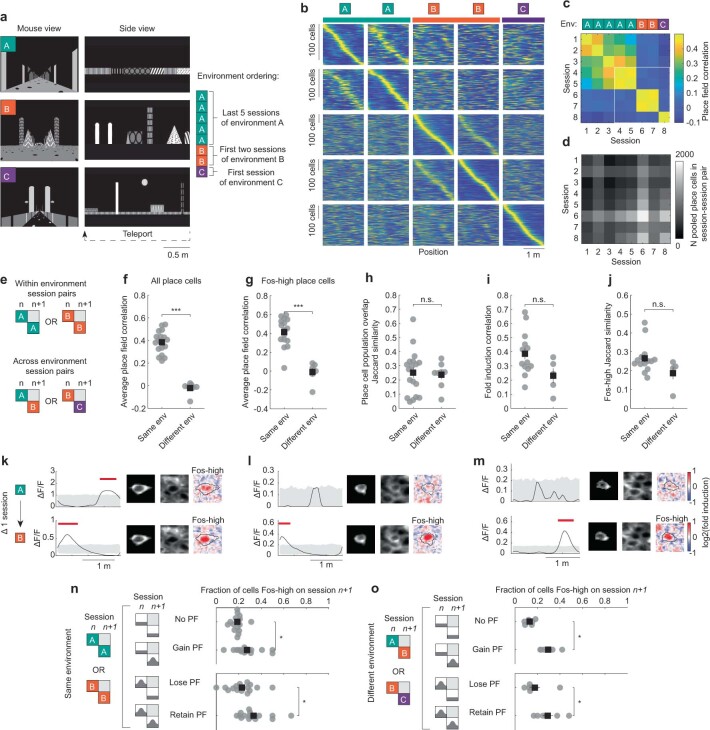
Place field remapping and Fos-induction across multiple virtual environments in the absence of a task. **a**) Three different virtual environments used to examine place field responses in the no-task condition. Mice were familiar with environment A before imaging began. **b**) Examples of place field remapping across environments. Each heatmap (25 heatmaps in total) shows the spatially binned activity of multiple cells, where each row is a cell. Each cell’s spatially binned activity is min-max normalized for the purposes of visualization. In the first row of five heatmaps, the same cells are shown in the same order in each column, ordered by their preferred position in the first environment A session. The second row is sorted by the second environment A session and so on. Each column in the grid corresponds to a different session. For each row in the grid, only cells with significant place fields on the sorting session and that are present in all sessions are shown. **c**) Average place field correlation across sessions. For each cell, only session-session pairs where at least one of the sessions has a significant place field are included in the analysis. The number of cells that went into each session-session comparison are shown in (**d**). Cells are pooled across 5 mice. **d**) Number of cells that went into each datapoint in (**c**). **e**) Schematic of session-session pairs analyzed in (**f**–**j**) within environments and across environments. To avoid within environment and across environment pairs being separated by different amounts of time, only consecutive session-session pairs were considered. **f**–**j**) Similarity metrics for place maps, place cell identity, and Fos-induction for session-pairs in the same environment or different environments. Same environment vs different environment: p = 7.3 x 10^−11^, n = 18 (same), 8 (different) pairs (**f**); p = 1.3 x 10^−6^, n = 16, 7 (**g**); p = 0.81, n = 18, 8 (**h**); p = 0.051, n = 14, 5 (**i**); p = 0.067, n = 14, 5 (**j**); two-sample two-tailed *t*-test. Black squares and error bars denote mean ± s.e.m. **k**–**m**) Three example cells tracked across environment switches. For each cell, the spatially binned activity is plotted (black line) along with the 1^st^ and 99^th^ percentiles of the shuffle distribution (gray). Red markers indicate significant place fields. **n**) Fraction of cells that are Fos-high on session n+1 out of cells that either continue to have no place field, gain, lose, or retain a place field within the same environment (only adjacent sessions are included). No PF vs Gain PF: p = 0.013, n = 16 session pairs. Lose PF vs Retain PF: p = 0.034, n = 16 session pairs. Paired-sample two-tailed *t*-test. Black squares and error bars denote mean ± s.e.m. **o**) Fraction of cells that are Fos-high on session n+1 out of cells that either continue to have no place field, gain, lose, or retain a place field across different environments. No PF vs Gain PF: p = 0.015, n = 6 session pairs. Lose PF vs Retain PF: p = 0.014, n = 6 session pairs; paired-sample two-tailed *t*-test. Black squares and error bars denote mean ± s.e.m. Source data

Second, we reasoned that, if Fos-high cells are related to place codes, then Fos induction should track the identity of place cells across different environments^18^. As expected, when mice were moved to virtual environments with distinct visual cues, the place code remapped^37^, including in Fos-high place cells (Extended Data Fig. 8a–g). During remapping, a cell’s Fos induction in an environment was related to whether the cell had a place field (Extended Data Fig. 8l,m). Of cells without a place field in one environment, those that gained a place field in a second environment were more likely to have high Fos induction in the second environment than those that continued to lack a place field (Extended Data Fig. 8o, top). Additionally, of cells with a place field in one environment, those that lost their place field in a second environment were less likely to have high Fos induction in the second environment than cells that had a place field in both environments (Extended Data Fig. 8o, bottom). Thus, Fos-high cells remapped in a manner consistent with place cells, and Fos induction tended to track the presence of a place field, even across multiple environments.

Together, these results demonstrate that cells with strong Fos induction are functionally distinct from neighbouring cells that have weak Fos induction. Fos-high cells are more likely to have place fields, have more reliable trial-to-trial activity and have higher spatial information at the population level. Moreover, their place field distribution is relatively uniform, in line with Fos-high cells encoding position rather than reward or valence^38^.

## A causal role for Fos in spatial coding

Given the strong correlation between Fos expression and spatial coding, we wondered whether Fos expression may causally shape CA1 activity and place codes. The place cell activity we measured on each day arose before Fos induction on that day, suggesting that the more reliable and spatially informative activity seen in Fos-high place cells may drive induction of Fos expression. However, we noticed that from session to session a similar set of cells had high Fos induction^39^ (Extended Data Fig. 6i–k), raising the possibility that Fos induction from the previous day could contribute to the reliability and accuracy of spatial coding in place cells on the subsequent session.

**Fig. 3:**
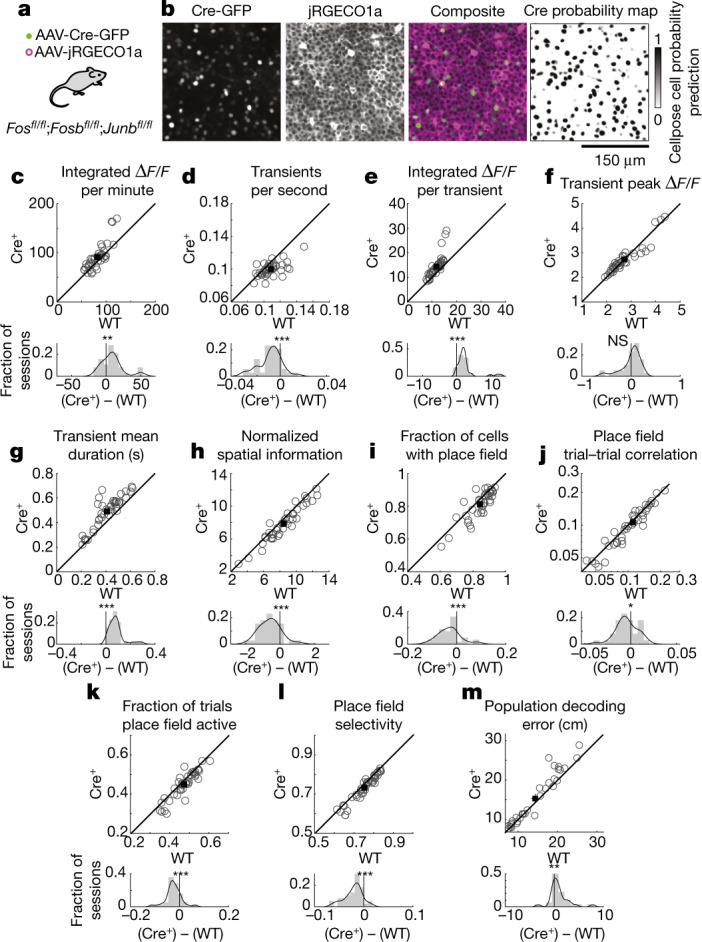
Causal effects of Fos family transcription factors on CA1 activity and place coding properties. **a**, *Fos*^*fl*/*fl*^;*Fosb*^*fl*/*fl*^;*Junb*^*fl*/*fl*^ mice were transduced with two AAVs to express jRGECO1a (high density) and Cre (low density). **b**, Representative two-photon images for a zoomed-in portion of the full imaging field of view. **c**–**n**, Properties for simultaneously imaged Fos-KO (Cre^+^; *y* axis) and wild-type (WT; *x* axis) populations. Top, each circle indicates the mean across neurons for one session. Bottom, difference histogram (grey) with kernel density estimation (black). Two-sided paired-sample *t*  test: *P* = 0.0014 (**c**), *P* = 4.9 × 10^−6^ (**d**), *P* = 2.7 × 10^−6^ (**e**), *P* = 0.96 (**f**), *P* = 6.6 × 10^−10^ (**g**), *P* = 8.6 × 10^−7^ (**h**), *P* = 0.00018 (**i**), *P* = 0.025 (**j**), *P* = 1.6 × 10^−5^ (**k**), *P* = 1.6 × 10^−8^ (**l**). *n* = 39 sessions for 6 mice. **m**, Scatterplot and difference histogram as in **c**–**l** showing the decoder error for Fos-KO and wild-type populations on individual sessions. *P* = 0.0095, two-sided paired-sample *t* test. *n* = 32 sessions for 6 mice. NS, not significant; *, *P* < 0.05; **, *P* < 0.01; *** *P* < 0.001. Source data

We therefore tested whether Fos expression has a causal role in regulating hippocampal place codes. Previously, we have shown that, of the seven partially redundant members of the Fos family of transcription factors, Fos, FosB and JunB are substantially more inducible in the hippocampus, and removal of these three members effectively disrupts the function of the Fos transcription factor complex^24^. We therefore used a triple-conditional knockout mouse line to ablate Fos, FosB and JunB and limited this knockout to only a small fraction of imaged neurons. Specifically, we injected a low titre of adeno-associated virus (AAV) encoding Cre recombinase fused to GFP into CA1 of *Fos*^*fl*/*fl*^;*Fosb*^*fl*/*fl*^;*Junb*^*fl*/*fl*^ mice (Fig. 3a,b). We identified knockout cells (hereafter referred to as Fos-KO cells) on the basis of appreciable Cre expression measured by GFP fluorescence. We have previously shown^24^ that expression of Cre, but not a catalytically inactive control, leads to excision of all three genes, permitting identification of effects specific to the disruption of Fos function. Approximately 20% of cells were Fos-KO cells (mean, 22.5%; range, 11.2–31.5%). We chose to disrupt the function of Fos in only a subset of cells because it is advantageous to compare Fos-KO cells to neighbouring wild-type cells side by side in the same session, thus controlling for any variability across sessions and mice. Moreover, this approach allows for identification of the cell-autonomous effects of Fos and eliminates confounds of network-level effects that may arise from global disruption of Fos.

**Extended Data Fig. 9:**
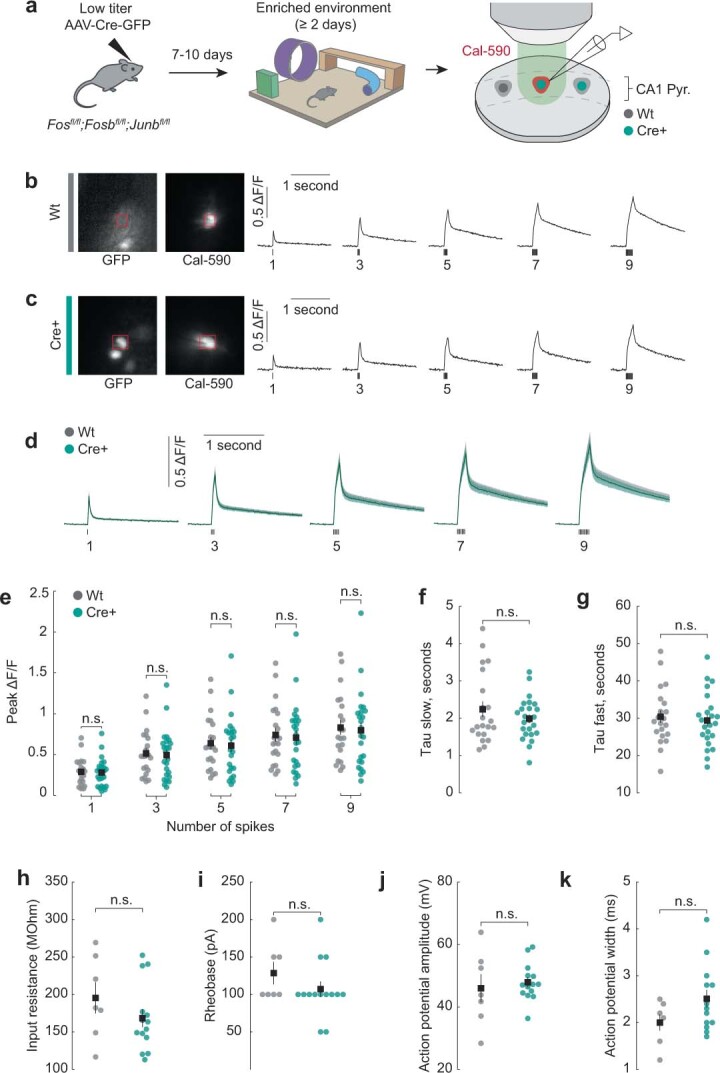
Spike-evoked calcium influx and electrophysiological properties of Fos-KO and wildtype neurons. **a**) Schematic of the experiment. Fos^fl/fl^;Fosb^fl/fl^;Junb^fl/fl^ mice were transduced with AAVs to express Cre-GFP in CA1 of the hippocampus (low density). Mice were exposed to an enriched environment for at least two days. **b**–**c**) Left, example images and ∆F/F fluorescence traces for Cre+ (Fos-KO) and Cre- (Wt) cells. Red boxes are somatic ROIs used to extract fluorescence traces. Right, example calcium imaging traces. Black ticks mark evoked spikes, numbers indicate number of spikes in a 50 Hz train. **d**) Average spike-evoked ∆F/F fluorescence for 1, 3, 5, 7, and 9 action potentials. Wt: n = 21 cells, 6 mice. Cre+: n = 24 cells, 6 mice. Mean ± s.e.m. across cells. **e**) Quantification of peak calcium influx. Wt vs. Cre+: 1 AP p = 0.87; 3 APs p = 0.82; 5 APs p = 0.78; 7 APs p = 0.79; 9 APs p = 0.79; two-sample *t*-test. Wt: n = 21 cells, 6 mice. Cre+: n = 24 cells, 6 mice. Black squares and error bars denote mean ± s.e.m. **f**–**g**) Decay kinetics quantified with double-exponential fit. Wt vs. Cre+: tau slow, p = 0.28; tau fast, p = 0.65; two-sample *t*-test. Wt: 21 cells, 6 mice. Cre+: 24 cells, 6 mice. Black squares and error bars denote mean ± s.e.m. **h**–**k**) Quantification of electrophysiological properties. Wt vs. Cre+: input resistance, p = 0.24; rheobase, p = 0.25; AP amplitude, p = 0.62; AP width, p = 0.10; two-sample *t*-test: Wt: n = 7 cells, 1 mouse. Cre+: n = 14 cells, 1 mouse. Black squares and error bars denote mean ± s.e.m. Source data

We observed significant differences in the neural activity of Fos-KO cells relative to their wild-type neighbours. Fos-KO cells had fewer calcium transients per second (Fig. 3d) but higher integrated calcium levels and longer calcium transient durations (Fig. 3c,e,g), indicating that Fos has a cell-autonomous role in altering neuronal activity levels. Other aspects of activity in Fos-KO cells were similar to those in wild-type cells, such as the peak amplitude of transients (Fig. 3f). In brain slice experiments, Fos-KO and wild-type cells had similar calcium influx per spike along with similar calcium transient kinetics and intrinsic electrophysiological properties, indicating that Fos is not involved in regulating these cellular properties (Extended Data Fig. 9). Thus, Fos-KO cells have altered calcium transients during the navigation task but normal calcium influx per spike, suggesting that Fos-KO cells have different numbers or patterns of spikes relative to wild-type cells. Although our experiments lack the resolution to determine what these differences in spiking are, they nevertheless demonstrate that disruption of Fos has specific effects on a cell’s activity in mice during navigation.

**Extended Data Fig. 10:**
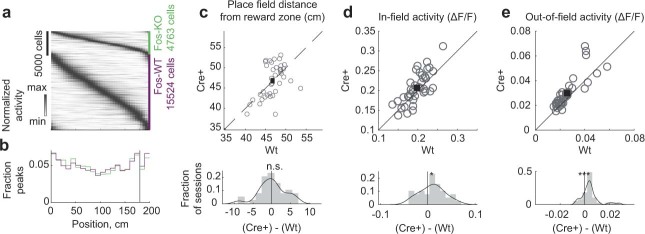
Fos-KO and Fos-WT place field distribution. **a**) Normalized activity of Fos-KO (top) and Wt (bottom) place cells as a function of track position. **b**) Histogram of peak location for Fos-KO (green) and Wt (purple) cells. Vertical gray line denotes start of reward zone. **c**) Top: Distance of place fields from the reward zone for simultaneously recorded Fos-KO (Cre+) and wild-type (Wt) cellvs. Each circle indicates the mean across cells for one session. Bottom: Histogram of the differences between Cre+ and Wt values in the above scatter. p = 0.85; two-sided paired-sample *t*-test. n = 39 sessions, 6 mice. **d**–**e**) Average in-field and out-of-field activity for simultaneously recorded Fos-KO (Cre+) and wild-type (Wt) place cells, plotted as in (**c**). p = 0.03 (**d**), p = 0.0007 (**e**); two-sided paired-sample *t*-test. Source data

We also observed significant differences in the place coding of Fos-KO and wild-type cells. Fos-KO cells had activity that was significantly less spatially selective than that of nearby wild-type cells, observed as lower spatial information in Fos-KO cells (Fig. 3h). In addition, Fos-KO cells were less likely to have a place field (Fig. 3i). Of the cells that had place fields, Fos-KO cells exhibited less reliable activity from trial to trial, measured as the correlation in activity between trials, and had activity in their place fields on a smaller fraction of trials (Fig. 3j,k). These cells also had higher activity outside their place field that resulted in decreased spatial selectivity when compared with their wild-type neighbours (Fig. 3l and Extended Data Fig. 10d,e).

Given the decreased reliability across trials, lower spatial selectivity and smaller fraction of cells with place fields in the Fos-KO population, we reasoned that Fos-KO cells might form less accurate spatial maps than their wild-type neighbours. We tested how well we could predict a mouse’s spatial location from the activity of equally sized populations of Fos-KO and wild-type cells. The accuracy of decoding the mouse’s location was lower when using the activity in the Fos-KO population than when using that in the wild-type population (Fig. 3m).

Together, these results point towards a causal role for Fos in shaping hippocampal function during spatial navigation. However, we note that Fos is not required for the expression of place fields, given that many Fos-KO cells had place fields. This finding might be expected because place fields arise within minutes in an environment^40^, which is faster than Fos induction and its downstream effects^11,23^. Instead, Fos has a causal role in regulating the activity properties of CA1 neurons and contributes to the expression of place codes with high reliability and accuracy.

The effects in Fos-KO cells were notably consistent across sessions, although the magnitude of the differences between Fos-KO and wild-type cells was relatively small and probably a substantial underestimate of the role of Fos in place cell coding. It would have been ideal to compare Fos-KO and wild-type cells using just the Fos-induced (that is, Fos-high) population. However, because of the already complicated loss-of-function mouse genetics, we were unable to additionally incorporate the *Fos*-transgenic reporter into these neurons to allow us to identify cells within the wild-type population with high Fos induction as in the experiments from Figs. 1 and 2. As a result, given that Fos induction tends to be relatively sparse, most of the wild-type cells probably would not have induced Fos in the first place. Thus, we could not directly compare the two relevant populations, namely wild-type cells with high Fos induction and Fos-KO cells that would normally have induced a high level of Fos. Together, these factors are expected to result in a substantial undermeasurement of the effect of Fos expression in place cell coding. It is therefore remarkable that we were able to observe consistent effects from loss of Fos from session to session, implying a robust role for Fos in hippocampal place codes.

## Stable place maps in Fos-high ensembles

Our results show that cells with high Fos induction form high-quality spatial maps that are regulated in part by Fos function. However, we do not know whether these Fos-expressing place cells have characteristics expected of an engram. An engram is predicted to be composed of neural ensembles that are made up of functionally connected and correlated neurons. Correlated activity among neurons could explain how activation of only a subset of neurons within an ensemble can trigger memory recall^4,7,8^. In addition, these ensembles are predicted to have stability across days to serve a role in memory^5,7,39^.

**Fig. 4:**
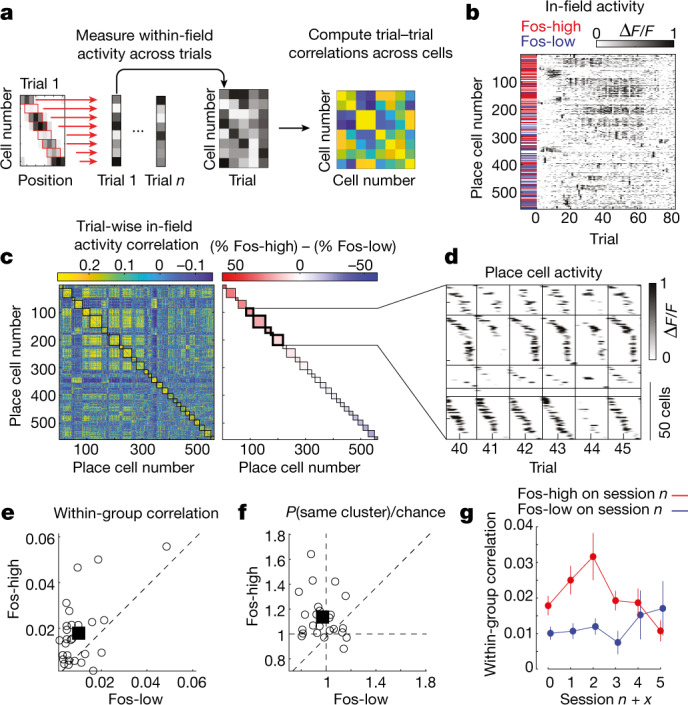
Correlation structure of Fos-expressing neurons. **a**, Method for computing trial-to-trial correlations between place cells independently of position selectivity. In brief, the mean activity in each cell’s place field was used to create an activity vector for each trial (left), and these were assembled into a matrix (middle). The correlations of average in-place-field activity across trials was computed for each cell pair (right). **b**, Mean in-place-field activity across neurons and trials for a representative session. For each trial, a cell’s mean in-place-field activity was calculated, resulting in one value per cell per trial. **c**, Left, matrix of correlations between neurons for their trial-wise place field activity, calculated using data arranged as in **b**. Right, clusters sorted by the difference in the percentage of Fos-high and Fos-low neurons in the cluster. Colour keys decrease from left to right to match ordering of the population. **d**, Representative activity of cells in a subset of clusters, shown as smoothed spatially binned activity for each cell across multiple trials. Cells within each cluster were ordered by their place field location. **e**, Within-group correlations for Fos-high and Fos-low cells. Correlations were calculated for a pair of cells as their trial-to-trial in-place-field activity. Circles correspond to individual sessions. *P* = 0.0024, two-sided paired-sample *t* test. *n* = 27 sessions for 6 mice. **f**, Probability that Fos-high (*y* axis) and Fos-low (*x* axis) cells belong to the same cluster relative to chance. Chance was obtained by permuting Fos induction labels. Clusters were determined as in **c**. *P* = 0.00074, two-sided paired-sample *t* test. *n* = 27 sessions for 6 mice. **g**, Within-group correlations across days for Fos-high and Fos-low cells. Data are shown as the mean ± s.e.m.; *n* = 27, 21, 17, 14, 8 and 5 sessions, from left to right. Source data

We first asked whether Fos-high neurons have activity that is highly correlated with the activity of other Fos-high neurons. Because a place code is made up of cells that are active in a sequence across the track, we wanted to measure whether Fos-high neurons tend to have their highest activity on the same trials as other Fos-high neurons, regardless of the location of their place fields. We therefore computed correlations between place cells using the mean activity within their place fields on every trial, instead of using the raw time series of their activity (Fig. 4a,b). This approach quantifies the degree to which a pair of place cells have shared trial-by-trial fluctuations in their place field activity, even if their place fields are at different locations. It does not consider temporal correlations of the raw traces, which would instead reflect neurons tending to be active at similar locations. Within a session, Fos-high neurons had high correlations with other Fos-high neurons, compared with the correlations among Fos-low cells (Fig. 4e). Notably, this correlation structure was preserved at similar levels for several days following Fos induction (Fig. 4g).

In addition, using the entire population of neurons, we clustered cells on the basis of their correlations with other cells, as calculated above (Fig. 4c). Each cluster was a group of cells that had highly correlated activity with one another, regardless of their place field locations. Fos-high neurons were more likely to reside in the same cluster than Fos-low neurons, further indicating that Fos-high cells are a population with substantially higher activity correlations with one another (Fig. 4f). Together, these results demonstrate that Fos-high cells form ensembles with highly correlated activity.

We examined the arrangement of cells that participated in these ensembles (Fig. 4d). We reasoned that groups of neurons with high activity correlations may make up an ensemble specific to a particular location on the track. Instead, clusters of cells with high correlations consisted of place cells with fields separated in space and that notably spanned the length of the track. Therefore, the clusters of Fos-high neurons form ensembles that are configured as place cell sequences and thus could serve as a spatial map of the entire track.

**Fig. 5:**
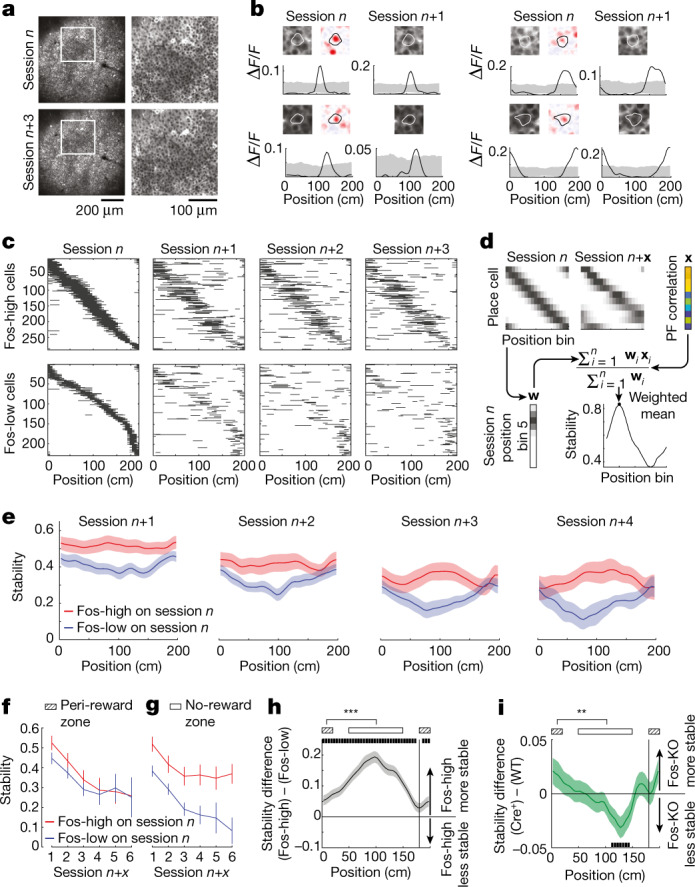
Fos expression is associated with higher spatial map stability. **a**, Example field of view, with a zoomed-in image (right). **b**, Representative place cells imaged on consecutive sessions. Top, jRGECO1a and fold-induction images. Bottom, average activity as a function of track position. Shading indicates the 1st and 99th percentiles for permuted activity relative to position. **c**, Example of place field stability for a subset of Fos-high (*n* = 289) and Fos-low (*n* = 230) place cells in one mouse. Black lines denote significant spatially binned activity in that portion of the track. A cell might appear more than once if it was induced on multiple sessions. Same ordering of cells across sessions. **d**, Schematic of stability calculation. For every pair of sessions, the correlation *x* between the spatially binned activity vectors is computed for each cell. Stability is the weighted mean of *x* in each bin, where weights *w* are proportional to the normalized activity of cells in that bin (Methods). **e**, Average stability maps for Fos-high and Fos-low populations. For each session-to-session comparison, Fos-high and Fos-low groups were determined on the basis of induction on session *n*. Data are shown as the mean ± s.e.m. *n* = 21, 17, 14 and 8 session-to-session comparisons, from left to right. **f**,**g**, Average stability in the peri-reward zone and no-reward zone of the track (zones indicated in **h**,**i**) across sessions. Data are shown as the mean ± s.e.m. *n* = 21, 17, 14, 8, 5 and 3 session-to-session comparisons, from left to right. **h**,**i**, Difference in stability between Fos-high and Fos-low (**h**) and Fos-KO (Cre^+^) and wild-type (WT) (**i**) cells as a function of track position. Black markers indicate bins where *P* < 0.05. ***P < 0.001 (**h**) and **P = 0.005 (**i**) for comparison of the mean stability difference in peri-reward and non-reward zones, two-sided bootstrap permutation test (1,000 shuffles). Data are shown as the mean ± s.e.m. *n* = 68 (**h**) and 114 (**i**) session-to-session comparisons. Source data

To examine the stability of place fields across days, we tracked the same cells across multiple sessions, with sessions typically occurring on consecutive days (Fig. 5a,b). The place fields in Fos-high cells tended to be stable and present at similar locations on subsequent sessions, whereas the place fields in Fos-low cells tended to disappear or shift in their location across sessions (Fig. 5b,c). To quantify this observation, we computed spatial stability maps based on the across-day similarity of place fields (Fig. 5d). Fos-high neurons exhibited higher stability in their place code (Fig. 5e). Fos-high cells had similar stability across all locations on the track, whereas Fos-low cells were most stable near the reward zone (Fig. 5e). As a result, the largest difference in stability between Fos-high and Fos-low cells was in the unrewarded part of the track (Fig. 5f–h). This result is consistent with our earlier finding that Fos-high neurons more uniformly tile the track than Fos-low cells, which are enriched at the reward site (Fig. 2e).

In addition, the place fields of Fos-KO cells were significantly less stable than those of their wild-type neighbours (Fig. 5i). The lower stability in Fos-KO cells was specific to the unrewarded parts of the track (Fig. 5i), in line with the finding that the biggest difference in stability between Fos-high and Fos-low cells was at locations away from the reward site. Together, the results for Fos-high and Fos-KO cells show that Fos expression is associated with stable maps that are mostly spatially unbiased and span the entire track. By contrast, cells with low Fos induction or disrupted Fos function have stable place fields only near the reward site and thus do not form stable maps of the entire track. Therefore, these results define a strong relationship between Fos expression and the neural ensembles that make up stable and spatially uniform maps. While we did not test the necessity or sufficiency of these neurons in spatial memory, the organization of these ensembles as sequences of place cells that are stable across days matches the expected form of a neural population underlying an engram for spatial memories.

## Discussion

Our results present multiple lines of evidence establishing a relationship between Fos-expressing cells and hippocampal place codes and further define a role for Fos in aspects of this relationship. First, Fos-high cells have higher place field prevalence and more reliable place field activity from trial to trial on a given day than Fos-low cells and thus allow for more accurate decoding of a mouse’s location in its environment. Second, knocking out Fos function results in a degraded spatial map compared with neighbouring wild-type cells owing to a lower prevalence of place fields and less reliable activity from trial to trial. Third, Fos-high cells have higher stability in their place fields across days, and Fos-KO cells have less stable place fields across days. Fourth, the place fields in Fos-high cells tile the track uniformly, whereas Fos-low cells are enriched at the reward zone. Fifth, Fos-high cells have a stronger relationship to place coding than Fos-low cells even in environments without rewards or a task and during remapping in different environments. Sixth, Fos-high cells, but not Fos-low cells, form ensembles of neurons that are co-active on the same sets of trials, and these ensembles are arranged as sequences of place cells.

The characteristics of Fos-expressing cells we describe here point to a link between the cognitive map theory of hippocampal function^1,2^—a framework for describing how spaces are encoded by the activity of neurons—and contextual memory encoding, which the hippocampal engram has been most shown to support^7–9^. Fos expression, which is often used to define hippocampal engrams, seems to be specifically related to the encoding of place, rather than the encoding of features of the animal’s behaviour or valence (in this case, reward). In addition, the correlated activity among Fos-high neurons is suggestive of an ensemble of functionally interconnected cells, a feature of engrams that has been proposed^4^ to support recall of an entire environment or experience from the synchronous activation of just a few neurons. Finally, the stable and spatially uniform maps formed by Fos-expressing neurons could act as a contextual reference for memory formation and recall. Because the same cells induce Fos on repeated days, these cells are involved in multiple experiences that take place in the same context^18,41^. Such a map that is invariant to specific experiences and stable over time could act as a general spatial reference participating in multiple episodic memory engrams that share a common environmental context.

A previous study proposed that Fos-expressing neurons encode context and are unrelated to spatial maps in novel environments^10^. This study found that Fos-expressing cells had less spatial information per spike than non-Fos-expressing cells and were more likely to remap following re-exposure to a novel environment^10^, which is different from what we report here. While our work and this previous study used different methods to explore spatial behaviours, to identify Fos-expressing neurons and to measure neural activity, these differences are unlikely to explain the results. For example, studying place cells with calcium imaging and virtual reality, as we did here, is a well-established approach that has been shown to find similar place cell properties as other methods^42–44^. Instead, a key difference between the studies is that the previous work focused on Fos-expressing cells in novel environments, whereas we studied these cells in familiar environments. Fos is induced more broadly in novel environments^45^, and it is likely that novelty-related cues, such as new odours, sounds, sights and textures, strongly drive induction in these environments. As environments become familiar with repeated exposures, induction of Fos by non-spatial novelty-related factors declines, possibly leaving spatial factors as a primary driver of Fos expression. In this case, because place cell firing induces Fos expression and, in turn, Fos is necessary for creating a stable spatial map in familiar environments, as we showed here, this feedback loop may strengthen the place code and sharpen the link between Fos and place codes as environments become familiar. Indeed, the CA1 place code transitions from being unstable in novel environments to stable in familiar environments^46,47^. Thus, it is possible that Fos-expressing neurons encode the feature that is most relevant to the hippocampus, which may be context in novel environments and space in familiar environments. Finally, it is also possible that spatial codes and non-spatial contextual codes co-exist in Fos-induced neurons, as not all Fos-high neurons we recorded exhibited place fields.

Notably, the properties of our Fos-high ensembles are similar to those of place cells that participate in sharp-wave ripples^48^. For example, similarly to Fos-high cells, place cells that are modulated by sharp-wave ripples have fields that evenly tile the entire environment, are not biased towards the reward location and are stable over time^48,49^. These similarities suggest that our Fos-high ensembles may be involved in memory consolidation during quiet wakefulness or sleep, as sharp-wave ripple events occur during these periods and contain compressed replay or reactivation of place cell sequences critical for stable spatial memories^50^.

We were motivated to expand our investigations beyond only using Fos as an activity marker and aimed to identify a causal role for Fos in shaping hippocampal activity and place codes. We reasoned that, if Fos were implicated in this process, this could serve as a molecular inroad to begin to establish a mechanistic framework for understanding the cellular, synaptic and circuit bases of episodic memory regulation. In line with this idea, our results implicate Fos in shaping place codes but not in the initial establishment of place fields. We have recently shown that Fos, through a downstream effector, Scg2, affects long-lasting changes at distinct subtype-specific inhibitory synapses onto CA1 pyramidal neurons but does not appear to affect excitatory synaptic transmission onto these cells^24^. Overall, our results are consistent with previous work suggesting that place fields may initially form through excitatory synaptic mechanisms^51,52^, while inhibition is critical for modulating the firing of place cells that have already formed, including for suppression of out-of-field firing and regulation of cellular activity levels and timing^27,28^. Fos may therefore regulate place coding by setting up the appropriate sources and levels of inhibition required to maintain functional place cell networks. However, this is just one of many possible mechanisms by which Fos may shape neural activity in CA1. Future work will examine the role of Fos-mediated inhibitory plasticity directly in behaving animals and explore other potential pathways by which Fos can influence place cell function. In addition, it is likely that Fos acts in conjunction with other pathways, as expected for a process as complex as learning and memory of spatial maps. Although we did not evaluate the effect of Fos expression on behaviour here because we disrupted Fos function in only a sparse set of neurons, the degradation in the spatial code that we observed in Fos-KO cells is in line with our recent findings that knocking out Fos in a large fraction of CA1 cells impairs spatial learning in the Morris water maze^24^.

Beyond the relationships to place coding, our work investigates the activity patterns that drive Fos expression in the hippocampus during behaviour and examines a causal role for Fos in regulating the overall activity levels of neurons^10,17,53^. During spatial navigation, Fos is induced most strongly in cells with higher total calcium influx and long-duration calcium transients, and removal of Fos results in longer calcium transients. While these results may be due to differences in the underlying spiking activity, future work will be needed to characterize how the number, rate and pattern of spikes differ between these groups. It is possible that Fos operates in a negative-feedback loop to regulate a cell’s overall activity levels, whereby higher activity drives Fos induction and Fos then acts to restrict the type of activity that led to its induction.

Together, our study demonstrates a critical role for Fos-expressing cells in spatial coding, thus providing a framework by which to understand how hippocampal activity may support both spatial and contextual memories. We also demonstrate that Fos not only serves as a marker of active neurons, but also has an instructive role in shaping hippocampal activity and thus function. Future work will be critical to address the molecular, cellular and circuit mechanisms by which Fos sculpts place codes during spatial navigation as well as in other behavioural contexts.

## Methods

### Mice

All experimental procedures were approved by the Harvard Medical School Institutional Animal Care and Use Committee and were performed in compliance with the Guide for Animal Care and Use of Laboratory Animals. For muscimol inactivation experiments, data were collected from four adult wild-type C57BL/6J male mice (Jackson Laboratory, stock no. 000664). For Fos staining experiments, data were collected from four adult wild-type C57BL/6J male mice (Jackson Laboratory, stock no. 000664) and four Npas4-FH^24,54^ mice (Greenberg laboratory, Harvard Medical School), split evenly between the control and exposed conditions. Npas4 staining was not analysed. For Fos-GFP reporter imaging experiments, data were collected from four adult Thy1-jRGECO1a^55^ × B6.Cg-Tg(Fos-tTA,Fos-EGFP*)1Mmay/J double-transgenic male mice and seven B6.Cg-Tg(Fos-tTA,Fos-EGFP*)1Mmay/J transgenic male mice (Jackson Laboratory, stock no. 018306) injected with AAV (serotype 2/1) encoding a CAG-jRGECO1a red-shifted calcium indicator^56^. As the *Fos*-*GFP* transgene in these mice is not under the control of doxycycline and the Tet transactivator was not used, doxycycline was not administered in this study. GFP was localized to the nucleus owing to a nuclear localization sequence (M. Mayford, personal communication). For Fos-KO imaging experiments, data were collected from six adult *Fos*^*fl*/*fl*^;*Fosb*^*fl*/*fl*^;*Junb*^*fl*/*fl*^ male mice^24,57^. For all behaviour and imaging experiments, mice were at least 12 weeks old before the first data collection. For ex vivo experiments, data were collected from six *Fos*^*fl*/*fl*^;*Fosb*^*fl*/*fl*^;*Junb*^*fl*/*fl*^ male and female mice 4–6 weeks of age and six B6.Cg-Tg(Fos-tTA,Fos-EGFP*)1Mmay/J male and female mice 4–6 weeks of age.

### Virtual reality and behavioural hardware

We used a miniaturized modified version of a visual virtual reality system^58^ that has been described previously^42^. Head-restrained mice ran on an air-supported spherical treadmill that was constrained with a yaw and roll blocker to rotate only in pitch (forwards and backwards relative to the mouse’s body). Ball movement was detected by two optical sensors (ADNS-9800, Avago Technologies) connected to a Teensy-3.2 microcontroller (PJRC.com) mounted to a custom printed circuit board. Forward translation in the virtual environment was controlled by rotation of the ball, with velocity gain adjusted such that distance travelled in the virtual environment equalled the distance travelled on the surface of the ball. The virtual environment was back-projected (laser pico-bit projector, Celluon) onto a parabolic screen surrounding ~180 degrees of the mouse in azimuth, with a minimum screen distance from the mouse of approximately 5 inches. Designs for the virtual reality and behaviour hardware can be found at https://github.com/HarveyLab/mouseVR. Water rewards were delivered through a spout, with a solenoid valve controlling reward timing and quantity. Licks were detected by an electrical circuit triggered by contact with the lick spout.

### Virtual environment

The virtual track was constructed using the Virtual Reality Mouse Engine (ViRMEn)^59^ in MATLAB (Mathworks). The end of the track was continuous with its beginning, such that the track repeated with a circular topology. The walls of the track were tiled with textures to serve as visual landmarks. In the no-task and remapping experiments, two additional linear-track environments were introduced that were visually distinct with an inter-trial interval of 5 s.

### Behaviour task

Mice were transported from the housing facility in a light-blocked cart and kept in a dim experimental room throughout the day to minimize unintended Fos expression. Mice were trained to lick for water rewards in a hidden reward zone one-tenth the length of the track (20 cm). Before being exposed to the virtual environment, mice were habituated to the apparatus and trained to run and lick the water spout to receive rewards. Once mice transitioned into the virtual environment, the task contingency was fixed and water rewards were delivered after the first lick in the reward zone. Occasionally, manual rewards were delivered to ensure that the lick detection and reward delivery systems were working; trials with manual rewards were excluded from further analysis. In the final version of the behavioural task, mice were required to traverse the linear track and lick in a specific reward zone to receive water rewards^48,60,61^. Three trial types were present within each session: standard, crutch and probe. In standard trials (60–70% of trials), a water reward was delivered after the first lick in the reward zone. In crutch trials (20–30% of trials), a water reward was delivered as soon as the mouse entered the reward zone, regardless of licking behaviour. In probe trials (less than 10% of trials), no rewards were delivered, regardless of the mouse’s licking behaviour. Probe trials allowed us to assess licking and running behaviour in the absence of rewards. For standard and crutch trials, licks that occurred in the reward zone after the delivery of reward were deemed ‘consumption licks’ and did not contribute to measures of licking selectivity or numbers of licks. All other licks were considered ‘test licks’ (non-consumption licks). Muscimol inactivation experiments were performed in an earlier version of the task where the size of the reward zone was 40 cm rather than 20 cm.

### Remapping experiments

Remapping experiments were performed in the absence of a task. On each session, mice were exposed to one of three environments: A, B or C. Mice were only exposed to a single environment each day. Imaging sessions began after mice had already been exposed to environment A; thus, environment A can be considered ‘familiar’. After up to five sessions of imaging in environment A, mice were moved to environment B for up to two sessions and then to environment C for up to a single session.

### Behaviour analysis

To quantify the degree to which mice remembered and licked selectively near the reward zone, we compared licking in an area 10 cm immediately before the start of the reward zone (where mice exhibited anticipatory licking) to an equally sized area on the opposite side of the track (100 cm removed, not associated with reward). Licking selectivity was defined as the difference in the number of licks within each of these zones divided by their sum: (licks_pre-reward_ – licks_unrewarded_)/(licks_pre-reward_ + licks_unrewarded_). Licking selectivity ranges from −1 to 1, with 1 indicating licking only in the pre-reward zone and 0 indicating chance levels. All analysis was performed on stable performance sessions, defined as the first session in which mice reached a licking selectivity of 0.6 and any sessions thereafter. Learning sessions (Fig. 1c) were defined as the first three sessions in the environment, regardless of performance (not exclusive with stable performance sessions).

### Muscimol

To test whether hippocampal activity was required for memory of the reward location, we inactivated CA1 using bilateral injections of muscimol (Sigma). A group of four wild-type male mice was used for these experiments. Trained mice were injected on alternate sessions with either muscimol (1 ng nl^–1^ in extracellular saline) or saline for ten consecutive sessions. Craniotomies were made before experimental testing and covered with Kwik-Sil. On the days of testing, mice were briefly anaesthetized and injections were made bilaterally into CA1 ~1.4 mm below the dura. The injection pipettes were slowly retracted to minimize back-flow. Mice were returned to their home cage and allowed to recover for at least 90 min before behavioural testing. The injection volumes used were 500 nl (two sessions), 100 nl (four sessions) and 50 nl (four sessions). Only sessions containing at least ten trials with a lick were analysed, to exclude sessions where mice may have become unmotivated to attempt the task (five sessions excluded).

### Histology

Mice were anaesthetized with 10 mg ml^–1^ ketamine and 1 mg ml^–1^ xylazine in PBS through intraperitoneal injection. When fully anaesthetized, the animals were transcardially perfused with 5 ml of ice-cold PBS followed by 20 ml of cold 4% paraformaldehyde (PFA) in PBS. Brains were dissected and post-fixed for 1 h at 4 °C in 4% PFA, followed by three washes (each for 30 min) in cold PBS. Coronal sections (40 μm thick) were subsequently cut using a Leica VT1000 vibratome and stored in PBS at 4 °C. For immunostaining, slices were permeabilized for 30 min at room temperature in PBS containing 0.2% Triton X-100. Slices were blocked for 1 h at room temperature with PBS containing 0.2% Triton X-100, 2% normal donkey serum and 0.1% fish gelatin. Slices were incubated in primary antibodies diluted in blocking solution at 4 °C for 24 h: mouse anti-Fos (Abcam, ab208942; 1:1,000) or rabbit anti-Fos (Synaptic Systems, 226003; 1:3,000). Slices were then washed three times each with PBS for 10 min at room temperature, incubated for 2 h at room temperature with secondary antibodies conjugated to Alexa dye (Life Technologies; anti-rabbit Alexa 488 (A21206), anti-rabbit Alexa 555 (A31572), anti-mouse Alexa 488 (A21202) or anti-mouse Alexa 555 (A31570); 1:250) and washed three times with PBS. Slices were then mounted in DAPI Fluoromount-G (Southern Biotech) and imaged on a virtual slide microscope (Olympus, VS120). To quantify Fos expression across different brain regions, 200 μm × 200 μm non-overlapping regions of interest (ROIs) were manually selected tiling each of the four brain regions (dentate gyrus, CA1, retrosplenial area and primary somatosensory area), when present, in each slice. Slices that were excessively damaged, exhibited severe imaging artefacts or had extensive non-specific antibody labelling were excluded at this stage. The number of cells was then counted within these ROIs. Cells were counted in an average of 10.72 ROIs per brain region in each mouse (total of 343 ROIs). ROIs were selected pseudorandomly and presented to the experimenter during counting. The experimenter was blinded to mouse identity and experimental group during both ROI selection and cell counting.

### Surgery: virus injection

Virus injections were carried out in mice before they were put on a water schedule. Craniotomies were centred around 1.8 mm lateral to the midline (right hemisphere) and −2.3 mm posterior to bregma. The approximate locations of the three craniotomies were (1.55, −2.3), (1.93, −2.08) and (1.93, −2.52) mm (medial/lateral (ML) and anterior/posterior (AP) axes, respectively) from bregma. Virus injections were performed using bevelled glass micropipettes ~1.3 mm below the dura. For population imaging in Fos-TetTag mice, 60 nl of AAV2/1-CAG-jRGECO1a (1 × 10^11^ genome copies per ml) was injected at each site. For sparse Cre infection, 100 nl of a 1:1 mixture of AAV2/1-CAG-Cre-GFP (1 × 10^11^ genome copies per ml) and AAV2/1-CAG-jRGECO1a (1 × 10^11^ genome copies per ml) was injected into each of the three locations. No virus injections were performed in the double-transgenic (Fos-tTA,Fos-shEGFP and Thy1-jRGECO1a) mice.

### Surgery: cannula implantation

Cannula implantation for hippocampal imaging was performed on mice after water schedules had started, at approximately 90% of initial body weight. The hippocampal window and headplate implantation surgeries were carried out as described previously^43,62^. During the cannula implantation surgery, dental cement was used to attach a titanium headplate to the skull parallel to the surface of the hippocampal window.

### Two-photon imaging

Data were collected using a custom-built resonant-scanning two-photon microscope. An air-supported spherical treadmill was mounted on a three-axis translation stage (Dover Motion) to position the mouse with respect to the objective (Nikon ×16, 0.8 NA water immersion). Two-photon excitation of jRGECO1a was achieved using a mode-locked diode-pumped femtosecond laser at 1,040 nm (YBIX, Time-bandwidth) or 1,070 nm (Fidelty-2, Coherent). A Ti:sapphire laser (Coherent Chameleon Vision II) was used to deliver 920-nm pulsed excitation for GFP imaging. Imaging took place at either 920 nm for GFP or 1,040/1,070 nm for jRGECO1a. Emitted light was filtered and collected by a GaAsP photomultiplier tube (Hamamatsu). The microscope was controlled by ScanImage 2019 (Vidrio Technologies). Images were acquired at 30 Hz at a resolution of 512 × 512 pixels corresponding to a field of view of 448 × 448 μm^2^ for two mice and 768 × 768 μm^2^ for ten mice. To synchronize functional jRGECO1a imaging and behavioural data, the imaging frame clock and a subset of behavioural signals were recorded in pClamp (Molecular Devices) at 1 kHz. After recording, the full set of behavioural signals and task data collected in ViRMEn were synchronized with the imaging clock and downsampled to the imaging frame rate (30 Hz), using linear or nearest-neighbour interpolation when necessary. To measure either *Fos* promoter-driven GFP or Cre–GFP, image stacks were acquired centred in the *z* axis on the imaging plane. These volumes typically consisted of 40 planes (512 × 512 pixels, the same resolution as in jRGECO1a imaging) separated by 4 µm, with 200 frames per plane. Both green and red channels were acquired for post hoc registration of the volume to the functional imaging plane.

### Maintaining the same field of view within and across imaging sessions

We used a custom headplate holder designed for reproducible day-to-day mounting of the mouse on the ball. Before imaging, the cannula and window were cleaned using rounds of filtered water and gentle vacuum suction to remove fine dust and debris. The mouse was positioned under the objective, and the field of view was manually aligned with a reference image taken on day 1 of the experiment. To maintain a consistent axial plane during imaging, a subset of recently acquired frames were registered online to a reference stack to estimate displacements from the target plane. These results were plotted throughout the experiment to guide periodic small manual adjustments countering axial and lateral drift. Post hoc assessment of drift and image quality was performed by manually examining sped-up and downsampled movies of the entire experiment after motion correction^63^. Experiments with insufficiently stable imaging quality or imaging plane were excluded before the start of in-depth analysis.

### Motion correction

Motion correction of jRGECO1a calcium imaging movies was performed offline using a custom MATLAB pipeline (available at https://github.com/HarveyLab/Acquisition2P_class) as previously described^63,64^. Motion correction accounted for non-rigid deformations taking place on subframe, full-frame and minute-long timescales. GFP stacks were registered in MATLAB using non-rigid registration (NoRMCorre)^65^ for frames within each plane, followed by an fast Fourier transform (FFT)-based rigid registration across planes. Stacks were aligned to the imaging plane using the red channel, and GFP planes out of plane with the imaging target plane were discarded from further analysis.

### Fluorescence source extraction and classification

After motion correction, spatial footprints of fluorescence sources in calcium movies were identified using Suite2p^66^ (Python version, https://github.com/MouseLand/suite2p). The resulting sources were classified into two groups: putative cell body and non-cell body sources. Classification was performed using a simple convolutional neural network trained in MATLAB on manually labelled data, using a network architecture, hyperparameters and training procedure described previously^64^ with the exception of two output classes rather than three. Non-cell sources were discarded from further analysis.

### Fluorescence trace preprocessing

We monitored neural activity as calcium transients, which previous work has shown to be related to spiking in pyramidal neurons^34–36,56^, using the red-shifted calcium indicator jRGECO1a. Raw traces extracted by Suite2p were further processed as follows: a baseline fluorescence estimate was computed as the 30th percentile in a 60-s moving window. ∆*F*/*F* traces were computed by subtracting and dividing the raw trace by the baseline. For most analyses, significant transient traces were used^52,67,68^. Significant transients were identified using the following procedure, based on previous work^68^ (Extended Data Fig. 4). ∆*F*/*F* traces were standardized by subtracting the median and dividing by the standard deviation. Threshold levels in units of standard deviation (σ) were chosen between 1 and 4 in 0.2σ increments. For every threshold level *t*, putative transients were identified as positive samples exceeding that threshold. For each putative transient *n* frames in length, we estimated a false positive rate as the number of negative-going transients (<–*t*) with at least *n* frames divided by the number of positive-going transients with at least *n* frames. Transients with a false positive rate of less than 0.001 (0.1%) were considered to be significant at that threshold level *t*. Frames that were significant at at least one threshold level were considered significant in the final output (union across threshold levels). Finally, transients separated by less than two frames were merged, and transients less than two frames in duration were removed. The final significant transient traces are original zero-baseline ∆*F*/*F* traces, where all frames without significant transients were set to zero.

### Cross-day alignment of sources

For each mouse, a reference session was chosen for alignment (approximately halfway between the first and last days of imaging, to maximize similarity to other imaging sessions). All sessions were registered with the reference session using non-rigid alignment on the mean jRGECO1a images (NoRMCorre)^65^. Source masks were transformed using this alignment to place all source masks for a mouse into a common reference space. CellReg^69^ (a distance model) was used on these aligned images to match cell IDs across different sessions.

### Fos induction measurements

Changes in *Fos* promoter-driven GFP expression were measured using time-lapse two-photon imaging^29,70^. To compute the fold change in GFP fluorescence 2–4 h after the start of behaviour, the registered post-behaviour GFP image was divided by the pre-behaviour GFP image. To account for changes due to non-uniformities in brightness across the field of view, the resulting image was normalized by dividing by an estimated background image. The background image was obtained by two-dimensional median filtering the fold-induction image with a filter size of ~50 × 50 µm^2^, approximately 30 times the area of individual cell sources. This normalization accounts for changes in fluorescence that could be due to changing imaging conditions rather than changing GFP expression, including non-uniform changes across the field of view, and it assumes that GFP fluorescence is relatively sparse and primarily localized to the nucleus, assumptions that are well supported by the literature. Regions of the fold-induction image that contained artefacts due to errors in registration or large changes in imaging quality were manually excluded from further analysis. For each cell source, fold-induction values were determined by averaging the pixel values of the fold-induction image within a circular ROI centred on the cell body, 10 µm in diameter. Fos-high cells were defined as cells with the 20% highest fold-induction values on a given session, and Fos-low cells were defined as cells with the 20% lowest fold-induction values on a given session. To determine the time course of induction (Extended Data Fig. 2), the same procedure was used with the addition that, before computing the fold-induction images, all images in the time course were standardized using the following procedure. Each image was standardized by its median and median absolute deviation and then rescaled by a multiplicative factor *A* and additive offset *B*, where *A* was the median absolute deviation of pixel values across all images in the series and *B* was the median pixel value of all images in the series. The median and median absolute values were chosen under the same rationale and set of assumptions as described above. The recovered time course was consistent with previous studies using this, and other, Fos-GFP reporters^17,29,30^.

### Place cell definition

For all place field analyses, trials were only considered if they met the following criteria: at least three licks, duration between 4 and 60 s, no experimenter-triggered rewards and occurring before 1.2 ml of cumulative reward had been delivered. In addition, following convention in the field, place cell characteristics were computed using only frames during running, defined as when speed exceeded 5 cm s^–1^. To assess significant spatial tuning, the track was divided into 40 bins, each 5 cm in size. For each cell, the average activity within each bin was computed, and the resulting binned activity was smoothed with a Gaussian kernel with a standard deviation of 1 bin, or 5 cm, with circular padding, consistent with the circular topology of the track. Significant peaks in the spatially binned activity were determined by a shuffle test. On each shuffle (*n* = 1,000 shuffles), the behaviour was circularly shifted by a random number of frames and then divided into six blocks of roughly equal duration and the order of those blocks was randomly permuted. This procedure perturbs the relationship between neural activity and behaviour while maintaining the temporal and autocorrelation structure of the activity and behaviour. For each shuffle, the same spatial binning and smoothing were performed. Peaks in the true binned activity that exceeded the 99th percentile of the shuffle distribution for at least three consecutive spatial bins were deemed significant peaks. Apart from the lower bound on the size of significant fields (imposed to limit false positives), we remained agnostic to the shape, amplitude and number of fields: any cell with significant peaks is referred to as a place cell, with the significant peaks of that cell corresponding to its place field.

### Spatial information

In addition to defining place fields, we computed spatial information for each cell in the population, regardless of whether it had a significant field. Spatial information was defined as the mutual information *H* between neural activity and position, using the following formula:

$$H=\mathop{\sum }\limits_{i=0}^{n}{p}_{i}{a}_{i}{{\rm{\log }}}_{2}\frac{{a}_{i}}{a}$$

where *i* is the *i*th spatial bin, *a* is the overall mean activity, *a*_*i*_ is the mean activity in the *i*th bin and *p*_*i*_ is the fraction of time spent in the *i*th bin. For each cell, we computed the real value and the value for each of 1,000 shuffles (see ‘Place cell definition’). We report the normalized spatial information for each cell as the real information divided by the mean across shuffles^71^.

### Place field properties

The trial-to-trial correlation of a place cell was defined as the mean of off-diagonal elements in the *n* trial × *n* trial Pearson correlation matrix of a cell’s trial-wise spatially binned activity. Entries comparing pairs of trials that lacked activity were set to zero. To compute the fraction of trials in which a place field was active, trials with at least one significant transient within that cell’s place field were considered ‘active’. To compute the selectivity of place fields, we measured the average spatially binned activity within a cell’s significant fields (in-field activity) and the average spatially binned activity outside a cell’s significant fields (out-of-field activity). Selectivity was defined as the difference between these values divided by their sum: (in-field activity – out-of-field activity)/(in-field activity + out-of-field activity).

### Position decoder

Naive Bayes’ decoders were used to decode position^72,73^ from the activity of groups of neurons within each session. The decoder assumed Poisson firing and independence between neurons and adopted a uniform prior for all spatial bins. The inclusion criteria for frames used for either training or testing were the same as those used for place field calculation. Even-numbered trials were always used for training (computing place field activity templates) while odd-numbered trials were used for testing. Decoding error was defined as the absolute difference between the true spatial bin and the decoded bin, ranging between 0 and 100 cm owing to the circular structure of the track. For each frame, the posterior probability of the mouse being in a given position bin ‘pos’ was computed as

$$P({\rm{p}}{\rm{o}}{\rm{s}}|{a}_{{\rm{g}}{\rm{r}}{\rm{o}}{\rm{u}}{\rm{p}}})=C(\mathop{\prod }\limits_{i=1}^{N}{{f}_{i}({\rm{p}}{\rm{o}}{\rm{s}})}^{{a}_{i}}){e}^{-\tau {\sum }_{i=1}^{N}{f}_{i}({\rm{p}}{\rm{o}}{\rm{s}})}$$

where *a*_group_ is the activity of all cells in the group being used for decoding, *C* is the normalization constant, *τ* is the temporal bin size of one frame (one-thirtieth of a second), *N* is the total number of cells and for each cell *f*_*i*_(pos) is the spatially binned activity template and *a*_*i*_ is the activity on the frame. The bin with the maximum posterior probability was selected as the decoded position on that frame. To compare decoder performance across cell groups, equally sized groups were used for decoding. For Fos-GFP imaging experiments, a decoder was trained and tested on each induction decile group independently. Fos-low and Fos-high decoding performance was the average performance of deciles 1–2 and 9–10, respectively. To compare Cre^+^ and wild-type groups, the number of cells used for decoding was selected on the basis of the size of the smaller cell group, with a minimum of 10 cells up to a maximum of 100 cells used for decoding. Each group was then randomly downsampled to this number of cells, and the decoder was trained and tested using these equally sized populations. The downsampling, training and testing procedure was repeated 100 times, and decoder performance was averaged for each group across these repetitions. Sessions were only included if they contained at least ten cells in the smaller cell group. The code for the naive Bayes’ decoder was modified from code available on the Buzsaki laboratory’s GIT repository (https://github.com/buzsakilab/buzcode).

Many factors can contribute to variability in the mean decoding accuracy across different positions of the track, including running speed, the configuration of visual cues^74^ and behavioural variability. In particular, higher running speeds in the region of the track away from the reward zone, combined with the relatively slow kinetics of the calcium indicator, probably contributed to the increased decoding error there. We therefore focused on comparing groups of neurons recorded simultaneously and treated identically during analysis. Decoding analyses were always performed on size-matched subpopulations, and testing and training frames were identical for each subpopulation tested.

### Activity matching

For activity-matched analyses, activity matching was always performed between Fos-high and Fos-low neurons on a given session. Activity matching was carried out on integrated ∆*F*/*F* (henceforth *α*), which is proportional to mean ∆*F*/*F* activity. First, activity bin edges were determined by binning log_10_(*α*) from the entire population into ten bins (using MATLAB’s histcounts function). Log-transformed activity was chosen to determine bin edges because the raw activity distribution has a long positive tail that would otherwise lead to low binning resolution for the majority of *α* values and relatively sparse counts in the bins with higher *α* values. Within each activity bin, Fos-high and/or Fos-low cells were subsampled to equal amounts pseudorandomly using the following iterative procedure. Fos-high and Fos-low neurons within the bin were randomly drawn from either the most active or least active half (for that bin) depending on whether the mean *α* of all subsampled Fos-high neurons up to that iteration was higher or lower than the mean *α* of all subsampled Fos-low neurons. For example, if the mean subsampled Fos-high *α* was higher than the Fos-low *α*, the next iteration would draw from the least active Fos-high neurons in that bin and the most active Fos-low neurons in that bin. This procedure continued until either all the Fos-high or all the Fos-low neurons in that bin were drawn, whichever came first. This had the effect of not only matching the binned activity distributions to be identical, but also balancing mean activity between Fos-high and Fos-low populations.

### Classification of Fos-KO cells

After alignment across days, individual cells in mice with conditional Fos knockout were manually examined for Cre–GFP expression. Cre probability maps were produced from aligned Cre–GFP images using Cellpose^75^. Cells were manually examined taking into account their source mask as well as jRGECO1a, Cre–GFP and Cre probability images. Cells were manually labelled as Cre^+^ (Fos-KO), Cre^–^ (wild type) or ambiguous. Ambiguous cells were excluded from further analysis.

### Ensemble analyses

The following analyses were performed only on cells with significant place fields. To identify groups of place cells that had correlated activity across trials, independently of the positioning of their place fields in the track, we quantified the level of activity of each cell within its place field(s) on each eligible trial (see ‘Place field definition’) within the session: we refer to this as the trial-wise place field activation. Within-group correlation was quantified as the mean pairwise correlation of trial-wise place field activation within each group. To visualize and quantify group structure within the trial-to-trial variability in the population, we clustered cells with affinity propagation^76^, using as similarity their pairwise correlations in trial-wise place field activation (preference parameter, −1; max iterations, 5,000). To analyse within-group correlations across days, we examined pairs of sessions within the same animal separated by the variable ∆sessions. We will refer to the earlier session as the reference session and the subsequent session as the target session: for example, comparing sessions 3 and 4 corresponds to a ∆+1 pair, where the reference session is 3 and the target session is 4. Because we only considered pairs with a positive ∆ value, each unique pair of sessions was considered at most once in the analysis. For every pair of sessions with a positive ∆ value where the reference session had Fos induction data, the cell groups (Fos-high and Fos-low) were defined by induction quantile (top 20% and bottom 20%, respectively) on the reference session. We then computed the within-group correlation between these cells in the target session, as described above.

### Place field stability analyses

To analyse the relationship between Fos induction or Fos disruption and place field stability, we took the approach of comparing pairs of sessions separated by differing numbers of sessions within the same animal. For clarity, we will refer to one session as the reference session and the other as the target session, where the difference between them (∆sessions) was measured as the target minus reference. We only considered pairs with a positive ∆sessions value, such that any pair of sessions was considered at most once, and the target session always occurred after the reference session. Fos induction cell groups were defined by induction values on the reference session. For each pair of sessions, we examined the stability of cells that were members of a specific cell group (Fos-high, Fos-low, Fos-KO, Fos-WT), had significant place fields on the reference session and were present on both sessions. At least 20 cells within each group were required for a session pair to be considered for stability analysis. For each cell group within each valid session pair, we computed stability maps that described place field stability as a function of track position. First, we computed for each cell the Pearson correlation between spatially binned activity on the reference and target sessions, producing the place field correlation vector x. Stability at each bin *i* along the track was computed as a weighted mean of the vector **x**, where the weight vector **w** for bin *i* was the normalized activity of each place cell in that bin. Thus, if a place cell exhibits no activity in a particular bin, it will not contribute to the mean stability of that bin while it will contribute strongly to the stability of bins around the peak of its place field. Before computing the weights, the spatially binned activity of each place field was normalized to sum to one so that the contribution of each place field to the overall stability map was equal regardless of the cell’s activity level.

### Ex vivo whole-cell electrophysiology and calcium imaging

#### AAV injection (*Fos*^*fl*/*fl*^;*Fosb*^*fl*/*fl*^;*Junb*^*fl*/*fl*^ mice)

Before ex vivo electrophysiological recordings, *Fos*^*fl*/*fl*^;*Fosb*^*fl*/*fl*^;*Junb*^*fl*/*fl*^ mice were injected with AAV-Cre-GFP to conditionally disrupt the Fos complex in a sparse subset of CA1 neurons, as previously described^24^. Surgical preparation and procedures up to virus injection were carried out as described in the surgery methods for in vivo experiments. Mice at 3–4 weeks of age were anaesthetized by isoflurane inhalation and placed in a stereotaxic frame (Kopf, model 1900). A small craniotomy was made on the dorsal surface of the skull, and virus injection was performed bilaterally using bevelled glass micropipettes targeted to CA1 (ML: ±3.0 mm; AP: −2.4 mm; dorsal/ventral, DV: −2.8 mm). Approximately 1 μl of AAV2/1-CAG-Cre-GFP (titre of 1.751 × 10^11^ genome copies per ml) was injected at a rate of 150 nl min^–1^, after which the pipette was left in place for approximately 5 min to allow the virus to diffuse and pressure to dissipate before retraction. Mice were allowed to recover for at least 7 days to allow for virus expression and recombination to occur.

#### Acute slice preparation

Mice were placed in an enriched environment before recording (at least 3 h for *Fos* reporter mice and at least 2 days for *Fos*^*fl*/*fl*^;*Fosb*^*fl*/*fl*^;*Junb*^*fl*/*fl*^ mice). The enriched environment set-up consisted of a large opaque cage (0.66 m × 0.46 m × 0.38 m) containing an assortment of toys and objects such as a running wheel, tubes, ladders, platforms, huts and different kinds of animal bedding. Food pellets were scattered throughout to encourage exploration.

Acute slice preparation was carried out as previously described^24^. Mice aged 4–5 weeks were anaesthetized by ketamine/xylazine injection and transcardially perfused with ice-cold oxygenated (95% O_2_/5% CO_2_) choline-based artificial cerebrospinal fluid (choline-ACSF) consisting of (in mM) 110 choline chloride, 25 NaHCO_3_, 1.25 NaH_2_PO_4_, 2.5 KCl, 7 MgCl_2_, 25 glucose, 0.5 CaCl_2_, 11.6 sodium l-ascorbate and 3.1 sodium pyruvate. Transverse 300-μm hippocampal slices were prepared from both hemispheres using a vibratome (Leica, VT1000). The dorsal hippocampal slices were collected in a holding chamber containing oxygenated ACSF consisting of (in mM) 127 NaCl, 25 NaHCO_3_, 1.25 NaH_2_PO_4_, 2.5 KCl, 1 MgCl_2_, 10 glucose and 2 CaCl_2_. The slices were then incubated at 32 °C for 20 min followed by 30 min at room temperature before recordings. Recordings were carried out at room temperature. For all solutions, pH was set to 7.2 and osmolarity was set to 280–290 mOsm.

#### Ex vivo electrophysiology and calcium imaging

For whole-cell current-clamp recordings, a K^+^-based internal solution consisting of (in mM) 142 potassium gluconate, 4 KCl, 10 HEPES, 4 magnesium ATP, 0.3 sodium GTP, 10 sodium phosphocreatinine and 1.1 EGTA (pH 7.2, 280 mOsm) was used. To record calcium transients, cells were filled with the synthetic red calcium indicator Cal-590 dextran (molecular weight, 4,000) at a 100 μM concentration. In all recordings, neurons were held at −70 mV with patch pipettes made with borosilicate glass with filament (Sutter, BF150-86-7.5) with open pipette resistance of 2–4 MΩ.

Simultaneous electrophysiological recordings and calcium imaging were performed on an upright Olympus BX51 WI microscope with an sCMOS camera (Zyla 5.5 sCMOS, Oxford Instruments), ×60 water-immersion objective (Olympus Lumplan Fl/IR ×60/0.90 NA) and light-emitting diode (Excelitas XCite LED120). Electrophysiological data were acquired using Clampex 10.6 (Molecular Devices). Data were low-pass filtered at 4 kHz, sampled at 10 kHz with an Axon Multiclamp 700B amplifier and digitized with an Axon Digidata 1440A data acquisition system. Experiments were discarded if the holding current exceeded −200 pA or if the series resistance was greater than 30 MΩ. Image acquisition was performed using MicroManager. Timestamps of frame acquisition were recorded on Clampex to allow for synchronization of electrophysiology to calcium images.

To measure spike-evoked calcium influx, each protocol consisted of five sweeps each with 1, 3, 5, 7 or 9 current injection pulses. Current was injected with a 4-ms square-wave pulse every 20 ms to evoke 50-Hz spike trains. The amplitude of the current injection was set by the experimenter to be slightly greater than the minimum value required to reliably evoke spikes with a 4-ms pulse, measured in 50-pA increments. Sweeps in which the number of spikes did not equal the number of current pulses were excluded from analysis. Between one and five repetitions of the protocol were averaged together to produce the spike-evoked fluorescence for each cell.

To measure the intrinsic electrophysiological properties of cells, a current–voltage (*IV*) curve protocol was carried out in a subset of cells. This protocol consisted of 500-ms current injections between 150 and 600 pA in 50-pA increments. Steady-state voltage used to construct *IV* curves was computed as the average membrane voltage in the last 100 ms of current injection. Input resistance was computed from the slope of the *IV* curve on current steps before the first current step in which a spike was evoked. The membrane time constant was computed using a single exponential fit to the membrane voltage polarization in response to the 50-pA negative current injection, using MATLAB’s lsqcurvefit function. Action potential waveform properties were computed on the first evoked action potential in the protocol. Action potential width was measured as the full width halfway in amplitude between the action potential threshold and the action potential peak.

#### Ex vivo electrophysiology and calcium imaging analysis

All ex vivo electrophysiology and calcium imaging analyses were carried out using custom scripts in MATLAB. Calcium imaging data were synchronized with traces using frame exposure trigger signals collected in the same time base as intracellular voltage and current injection data. To extract fluorescence traces from each cell, one rectangular ROI was drawn over the soma of the cell and another larger rectangular ROI was drawn outside the soma to estimate background fluorescence. Pixels within these ROIs were averaged on each frame to yield raw somatic and background fluorescence traces. To compute ∆*F*/*F*, the background fluorescence trace was subtracted from the somatic trace to yield a fluorescence trace *F*. *F*_0_ was computed for each sweep as the average *F* in the 200 ms before current injection. ∆*F*/*F* was then computed for each sweep as (*F* – *F*_0_)/*F*_0._ Between one and five repetitions of the protocol were averaged together to yield a set of five sweep traces per cell corresponding to 1, 3, 5, 7 and 9 action potentials triggered at 50 Hz. We observed that the decay kinetics appeared to have two components: fast and slow. This is consistent with our imaging picking up a mixture of somatic calcium dynamics, which tend to be lower amplitude and slower, and dendritic calcium dynamics, which tend to be higher amplitude and faster. Nuclear calcium dynamics may also be present as the dye is not restricted from the nucleus. This is expected because epifluorescence imaging does not allow excitation to be primarily restricted to either the soma or the dendrite. Decay kinetics were therefore fit with a double-exponential decay using MATLAB’s lsqcurvefit function.

#### Data analysis, statistics and reproducibility

Data were analysed in MATLAB (2019a and 2021b) together with the DataJoint toolbox^77,78^ (version 3.3.2). No statistical method was used to predetermine sample size. Sample sizes in terms of mice, sessions and neurons are similar to those in other contemporary studies in the field^16^. For in vivo imaging experiments, comparisons were made between simultaneously recorded neurons within mice that were subjected to the same experimental conditions; randomization across subjects and blinding to experimental conditions were not necessary and did not take place during calcium imaging experiments or analysis. During manual classification of Cre^+^ cells, the experimenter did not have access to information about the activity or functional properties of the cells. For manual cell counting of labelling with anti-Fos antibody, the experimenter was blinded to mouse identity and experimental group during both brain region ROI selection and cell counting (see ‘Histology’). For hypothesis testing, we used permutation shuffle tests, two-sided paired-sample *t* tests and two-sided two-sample *t* tests, showing both individual data points and difference histograms when possible. In Fig. 1i, representative images are shown from one session of the 27 sessions in 6 mice that were analysed in Fig. 1j–n. In Fig. 3b, representative images (cropped from the full field of view for the purposes of visualization) are shown from one mouse of the 6 mice analysed in Fig. 3. In Fig. 5a, two representative fields of view are shown from one mouse of the 6 mice analysed in Fig. 5.

### Reporting summary

Further information on research design is available in the Nature Research Reporting Summary linked to this article.

## Online content

Any methods, additional references, Nature Research reporting summaries, source data, extended data, supplementary information, acknowledgements, peer review information; details of author contributions and competing interests; and statements of data and code availability are available at 10.1038/s41586-022-05113-1.

## Supplementary information

Reporting Summary

## Data Availability

Data are available from the corresponding authors on request. Source data are provided with this paper.

## Source data

Source Data Fig. 1
Source Data Fig. 2
Source Data Fig. 3
Source Data Fig. 4
Source Data Fig. 5
Source Data Extended Data Fig. 1
Source Data Extended Data Fig. 2
Source Data Extended Data Fig. 3
Source Data Extended Data Fig. 5
Source Data Extended Data Fig. 6
Source Data Extended Data Fig. 7
Source Data Extended Data Fig. 8
Source Data Extended Data Fig. 9
Source Data Extended Data Fig. 10

## Code availability

Code is available from the corresponding authors on request. 
