# Supplementary material for: Fos ensembles encode and shape stable spatial maps in the hippocampus
Source: Nature. 2022 Aug 24;609(7926):327–34. doi: 10.1038/s41586-022-05113-1 (PMC9452297; doi:10.1038/s41586-022-05113-1)
Supplement: Supplementary file 1 — Reporting Summary [file 41586_2022_5113_MOESM1_ESM.pdf]

Corresponding author(s): Christopher D. Harvey

Last updated by author(s): Jun 22, 2022

Reporting Summary

Nature Research wishes to improve the reproducibility of the work that we publish. This form provides structure for consistency and transparency in reporting. For further information on Nature Research policies, see our Editorial Policies and the Editorial Policy Checklist.

Statistics

For all statistical analyses, confirm that the following items are present in the figure legend, table legend, main text, or Methods section.

n/a	Confirmed	
☐	☒	The exact sample size (*n*) for each experimental group/condition, given as a discrete number and unit of measurement
☐	☒	A statement on whether measurements were taken from distinct samples or whether the same sample was measured repeatedly
☐	☒	The statistical test(s) used AND whether they are one- or two-sided<br>*Only common tests should be described solely by name; describe more complex techniques in the Methods section.*
☐	☒	A description of all covariates tested
☐	☒	A description of any assumptions or corrections, such as tests of normality and adjustment for multiple comparisons
☐	☒	A full description of the statistical parameters including central tendency (e.g. means) or other basic estimates (e.g. regression coefficient) AND variation (e.g. standard deviation) or associated estimates of uncertainty (e.g. confidence intervals)
☐	☒	For null hypothesis testing, the test statistic (e.g. *F*, *t*, *r*) with confidence intervals, effect sizes, degrees of freedom and *P* value noted<br>*Give P values as exact values whenever suitable.*
☐	☒	For Bayesian analysis, information on the choice of priors and Markov chain Monte Carlo settings
☒	☐	For hierarchical and complex designs, identification of the appropriate level for tests and full reporting of outcomes
☐	☒	Estimates of effect sizes (e.g. Cohen's *d*, Pearson's *r*), indicating how they were calculated

Our web collection on statistics for biologists contains articles on many of the points above.

Software and code

Policy information about availability of computer code

Data collection	Virtual reality experiments were constructed and run using VirMEn (2016-02-12; Princeton) in combination with custom code in MATLAB (2019a and 2021b; MathWorks) for behavioral data collection. Micro-controller code used for running the virtual reality rig is available here: https://github.com/HarveyLab/mouseVR. Imaging experiments utilized ScanImage (version 2019a; Vidrio Technologies) and pClamp 10.3.1.4 (Molecular Devices). Epifluorescence imaging utilized MicroManager (v 2.0-gamma). For ex vivo electrophysiology, Clampex 10.6 was used. For images of brain sections, virtual slide scanner (VS120) software by Olympus or confocal microscope Leica Microsystems LSM700 software by Zeiss were used.
Data analysis	Preprocessing of calcium movies was carried out using custom MATLAB (2019a and 2021b) code (https://github.com/HarveyLab/Acquisition2P_class) and Suite2P (https://github.com/MouseLand/suite2p, versions 0.6.16 and 0.10.2). Registration of imaging stacks was carried out using custom MATLAB (2019a and 2021b) code and NoRMCorre (https://github.com/flatironinstitute/NoRMCorre, v0.1.1). Cellpose (https://github.com/MouseLand/cellpose, v0.5) was used to assist manual identification of Cre+ cells. CellReg (https://github.com/zivlab/CellReg, v1.3.7) was used to align sources across days. Datajoint (https://github.com/datajoint, version 3.3.2) in MATLAB (2019a and 2021b) was used for parts of the analysis pipeline. All other analyses were performed in MATLAB (2019a and 2021b). Code for Bayesian decoding of position was adapted from https://github.com/buzsakilab/buzcode/blob/master/analysis/spikes/positionDecoding/placeBayes.m. Custom code will be made available upon reasonable request.

For manuscripts utilizing custom algorithms or software that are central to the research but not yet described in published literature, software must be made available to editors and reviewers. We strongly encourage code deposition in a community repository (e.g. GitHub). See the Nature Research guidelines for submitting code & software for further information.

Data

Policy information about availability of data

All manuscripts must include a data availability statement. This statement should provide the following information, where applicable:

- Accession codes, unique identifiers, or web links for publicly available datasets
- A list of figures that have associated raw data
- A description of any restrictions on data availability

The data that support the findings of this study are available from the corresponding author upon reasonable request.

Field-specific reporting

Please select the one below that is the best fit for your research. If you are not sure, read the appropriate sections before making your selection.

☒ Life sciences ☐ Behavioural & social sciences ☐ Ecological, evolutionary & environmental sciences

For a reference copy of the document with all sections, see nature.com/documents/nr-reporting-summary-flat.pdf

Life sciences study design

All studies must disclose on these points even when the disclosure is negative.

Sample size	No statistical methods were used to predetermine sample sizes. The original submission contained in vivo calcium imaging data from 6 Fos-GFP mice and 6 FFJ (Fos-KO) mice. During review, data were collected from an additional 5 Fos-GFP mice for the no-task condition. The number of mice and sessions were chosen to approximately match the original Fos-GFP dataset in size. Sample sizes in terms of mice and neurons are similar to other contemporary studies in the field (For example, see Robinson et al. Cell 2020, Yap et al. Nature 2021, Harvey et al. Nature 2009, Harvey et al. Nature 2012, Danielson et al. Neuron 2016).
Data exclusions	A small number of 2P imaging sessions were excluded due to instability in imaging quality as described in Methods. A small number of histology sections were excluded from analysis if they were damaged or had insufficient quality of antibody labeling, as described in Methods. All other experiments were analyzed, with inclusion criteria for specific analyses as described in Methods.
Replication	The original submission contained in vivo calcium imaging data from 6 Fos-GFP mice and 6 FFJ (Fos-KO) mice. These data were collected prior to analysis. During review, data were collected from an additional 5 Fos-GFP mice for the no-task condition. These data were subjected to the same analyses as in the original submission, and the results were consistent with the original dataset collected during task performance. These data are presented separately in Extended Data Figure 8. No subsequent experiments were performed to replicate other findings from the original submission.
Randomization	All mice were subjected to the identical behavioral paradigm and imaging setup. For Fos staining and histology experiments, mice were randomly assigned to two "cohorts", each of which underwent identical pre-training and habituation. On the day of testing, one cohort was exposed to the virtual environment as described in Methods.
Blinding	All subjects took part in the same behavioral task and experimental conditions, (with the exception of Fos histology experiment, detailed below) therefore blinding during experiments was not necessary. All analyses took place after initial manual screening of the data (i.e. for imaging quality and stability). For counting Fos+ cells (Histology, Methods), the experimenter was blinded to mouse identity and experimental group during both ROI selection and cell counting, as detailed in Methods.

Reporting for specific materials, systems and methods

We require information from authors about some types of materials, experimental systems and methods used in many studies. Here, indicate whether each material, system or method listed is relevant to your study. If you are not sure if a list item applies to your research, read the appropriate section before selecting a response.

Materials & experimental systems

n/a	Involved in the study	
☐	☒	Antibodies
☒	☐	Eukaryotic cell lines
☒	☐	Palaeontology and archaeology
☐	☒	Animals and other organisms
☒	☐	Human research participants
☒	☐	Clinical data
☒	☐	Dual use research of concern

Methods

n/a	Involved in the study	
☒	☐	ChIP-seq
☒	☐	Flow cytometry
☒	☐	MRI-based neuroimaging

Antibodies

Antibodies used	Primary antibodies: mouse anti-Fos (Abcam ab208942), rabbit anti-Fos (Synaptic Systems 226003). Secondary antibodies: rabbit Alexa 488 (Life Technologies A21206), rabbit Alexa 555 (Life Technologies A31572), mouse Alexa 488 (Life Technologies A21202), mouse Alexa 555 (Life Technologies A31570).
Validation	Mouse anti-Fos (Abcam ab208942) and rabbit anti-Fos (Synaptic Systems 226003) were previously validated according to datasheets provided by the manufacturers (Yap et al. Nature 2021).

Animals and other organisms

Policy information about studies involving animals; ARRIVE guidelines recommended for reporting animal research

Laboratory animals	Imaging and behavioral data were collected from adult wild-type C57BL/6J male mice, adult Npas4-FH male mice (Sharma et al., Neuron 2019), adult Thy1-jJRGECO1a x B6.Cg-Tg(Fos-tTA,Fos-EGFP*)1Mmay/J double transgenic male mice and B6.Cg-Tg(Fos-tTA,Fos-EGFP*)1Mmay/J transgenic male mice (Stock No. 018306, Jackson Laboratory), and adult Fos(fl/fl);Fosb(fl/fl);Junb(fl/fl) male mice (Vierbuchen et al., Molecular Cell 2017), as described in Methods. For in vivo and behavioral experiments, all mice were at least 12 weeks of age prior to first data collection. For ex vivo whole-cell electrophysiology, Fos(fl/fl);Fosb(fl/fl);Junb(fl/fl) male and female mice were injected at 3-4 weeks of age and recorded from at 4-6 weeks of age, B6.Cg-Tg(Fos-tTA,Fos-EGFP*)1Mmay/J male and female mice were recorded from at 4-6 weeks of age. Mice were housed in a 12 h: 12 h reverse light:dark cycle at an ambient temperature of 22 °C and ambient relative humidity of 50%.
Wild animals	This study did not involve wild animals.
Field-collected samples	There were no field-collected samples in this study.
Ethics oversight	All experimental procedures were approved by the Harvard Medical School Institutional Animal Care and Use Committee and were performed in compliance with the Guide for Animal Care and Use of Laboratory Animals.

Note that full information on the approval of the study protocol must also be provided in the manuscript.
